# Exploring Host-Microbiome Interactions using an *in Silico* Model of Biomimetic Robots and Engineered Living Cells

**DOI:** 10.1038/srep11988

**Published:** 2015-07-16

**Authors:** Keith C. Heyde, Warren C. Ruder

**Affiliations:** 1 Department of Biomedical Engineering and Mechanics, Virginia Polytechnic Institute and State University, Blacksburg, VA 24061; 2 Department of Biological Systems Engineering, Virginia Polytechnic Institute and State University, Blacksburg, VA 24061.

## Abstract

The microbiome’s underlying dynamics play an important role in regulating the behavior and health of its host. In order to explore the details of these interactions, we created an *in silico* model of a living microbiome, engineered with synthetic biology, that interfaces with a biomimetic, robotic host. By analytically modeling and computationally simulating engineered gene networks in these commensal communities, we reproduced complex behaviors in the host. We observed that robot movements depended upon programmed biochemical network dynamics within the microbiome. These results illustrate the model’s potential utility as a tool for exploring inter-kingdom ecological relationships. These systems could impact fields ranging from synthetic biology and ecology to biophysics and medicine.

An organism’s evolutionary fitness is determined by how well it utilizes environmental metabolites. For constituents of the microbiome – the microorganisms associated with the animal body – their environment is a product of their host’s physiology. Yet, these commensal microbes also play a critical role in governing the health and behavior of their hosts. The effects include impacting host metabolism^1^, perturbing host hormone regulation^2^ and changing the host’s affinity for disease^3^. These interactions can even regulate complex animal behavior. For example, recent studies found that commensal *Lactobacillus plantarum* can affect the mating behavior of their *Drosophila melanogaster* hosts^4^, and that microbiome density can directly influence anxiety, and by extension, motility^5^ in mice.

These correlations likely result from inter-kingdom communication through biochemical signaling^6^. Furthermore, these communication motifs are present as relationships between consortia of commensal microbes and their host, not merely as interactions between a single microbial species and its host. Microbial consortia – consisting of multiple species and intertwined biochemical networks^7^ – allow for network complexity, as do spatial variations in the host ecosystem, rendering host-microbiome interactions difficult to fully understand and model^8^.

Fortunately, a variety of scientific tools exist to help explore complicated biological systems. Synthetic biology has generated multiple tools that have been used to probe and program cellular behaviors^9^ over the past fifteen years. The field was launched in 2000 by reports of the first synthetic biological networks – or engineered gene circuits – that functioned as memory^10^ or oscillatory^11^ modules in cells. Inspired by electrical engineering, these circuits have expanded to include other modules such as logic gates^12^, timers^13^, counters^14^, and simple analog computers^15^. These behaviors are programmed into DNA and then uploaded into cells. The resulting synthetic networks can then interface with endogenous networks within the same cell, organism, or commensal host to reprogram behavior (Fig. 1).

Progress in synthetic biology includes the creation of genetic component libraries as well as computational tools, giving researchers the ability to simulate analytical models of cellular response *in silico* prior to wet-lab assembly^16^,^17^. As a result, researchers now have an ability to rationally design, model, and build networks to probe and control specific cellular behaviors, leading to potential therapeutic interventions and broader scientific discovery.

Synthetic biology’s techniques are performed with increasing robustness using a number of individual model species including *E. coli*. However, most naturally occurring bacteria live in communities, or consortia^18^ of multiple species. Despite recent studies successfully demonstrating how engineered consortia can behave as a predator-prey system^19^ or geospatially self-organize^20^, the genetic network complexity required for targeted consortia engineering is daunting^21^. Additional modeling and engineering approaches are needed to further explore the details of microbiome interactions.

While examining the physiology of commensal microbes is important, macro-scale host behaviors must also be better understood to elucidate host-microbiome interactions. Biomimetic approaches give us a robust toolset for analyzing animal behavior. For example, biomimetic robots have served as tools for exploring biomechanics ranging from snake locomotion^22^ to human balance^23^. These robots provide quantifiable, minimal systems representative of natural phenomenal and are useful for scientific inquiry. In addition to mechanics, robots can be used to study cognition. By programming robots with a minimal set of algorithms and subjecting them to complex environmental challenges, researchers have used biomimetic robots to understand how primitive life forms solve a wide range of problems despite simple neural architectures^24^.

**Model System: A Robotic Host with a Living Microbiome**

In order to explore host-microbiome interactions, we created an *in silico* model system that combined the tools of synthetic biology and biomimetic robotics to design, model and computationally simulate a hybrid robot-bacteria system (Figs. 1 and 2). First, we conceptualized a physical system that could be built, consisting of a mobile robotic platform endowed with the capacity to harbor and communicate with a living microbiome. We envisioned this system as consisting of three physical modules that could be built with existing technology (Figure S1). We then modeled and simulated these individual modules together to produce the complete system’s behavior. Our final *in silico* model system was able to replicate a range of different biological ‘host’ behaviors. Specifically, when we increased the complexity of gene circuit topologies in the living microbiome, unique robot behaviors were captured by this *in silico* tool.

**Model System: Physical Modules**

We envisioned our hybrid robot-microbiome system to be comprised of three physical subsystems, or modules. These modules would exchange information through chemical, optical, and electrical signals. The first module (Figure S1A) would be an engineered microbiome consisting of a living, synthetically engineered *E. coli* population. These bacteria would be engineered with gene circuits that drive fluorescent reporter expression (i.e., increases in green fluorescent protein (GFP) or mCherry – a red fluorescent protein – as shown in Fig. 2C). This engineered microbiome would be housed within the second module.

The second module would consist of a microfluidic chemostat^25^ (Figure S1B) that would be monitored by a miniature epifluorescent microscope^26^ (Figs. 2B and S1B). Cells would be well mixed and in exponential phase, similar to previous studies^19^,^27^,^28^. Finally, this module would include electronics that sense and process the light signal from the epifluorescent microscope (EFM) into an electronic EFM signal (Supplementary Table 1, Fig. 2C). This signal, hereafter referred to as the EFM signal, would be sent to the third module.

The third module would be the biomimetic robot host (Fig. 2E and S1C). This third module would use the EFM signal to activate simple motion subroutines (Supplementary Table 2, Figs. 2F and S1C). The sum of these simple behaviors would then emerge as more complicated robot behaviors (Fig. 1B,C,F). Figure 2 illustrates how information would flow between the three modules. Here, we demonstrate the system with a simple synthetic circuit in the living microbiome, in which lactose drives GFP expression. All three modules are described in greater detail in Supplementary Text S1.

**Model System: Computational Simulation of Proposed Physical Modules**

Next, we modeled and computationally simulated all three physical modules of the proposed robot-bacteria system (Figure S2 and Supplementary Text S1). The resulting complete system behavior was then simulated in a two-dimensional, virtual testing environment – or arena. This environment included stationary carbon source depots, which were conceptualized to contain inducers such as lactose or arabinose. These depots were conceptualized as prey for the robotic system. When a robotic system docked with these depots, it would capture the depot’s inducer carbon source and a potential fitness advantaged would be conferred to the host. The details of this ‘docking’ are described in Supplementary Text S1.

The robot’s behavior in this simulated arena served as the output of our simulation. By analyzing the effects of variations in genetic circuit topologies and circuit parameter space, we observed a clear ability for the microbiome to cause distinct behavioral regimes in its host.

## Results

### Environment Simulation

In order to observe emergent robotic behavior, we designed an environmental simulation scenario. This scenario placed a robot in a 20 m × 20 m virtual, two-dimensional (2D) arena with an initial position at the center of this square. The simulated arena was initialized with one lactose and one arabinose carbon depot at different locations within the arena. These depots would remain in their position until the robot’s resulting movements led it to reach and dock at a depot, thereby acquiring all of the stored lactose or arabinose. This docking would cause the lactose or arabinose from the depot to enter the onboard microbiome at a constant concentration and rate. After a given carbon depot was depleted, a new source appeared of the same inducer (i.e., lactose or arabinose), but at a different location. In order for computational and analytical simplicity, we modeled the carbon sources to appear on the vertices of a 10 m × 10 m square, centered on the robot’s initial position. Finally, we initialized the biochemical environment of the microbiome with a simulated injection of arabinose at time t = 0.

### Simulation: Balanced Toggle Switching in Engineered Microbiome and Robot Behavior

In order to test our hypothesis that programming commensal bacteria with engineered gene circuits can result in new emergent host behavior, we simulated different engineered gene circuits in our model system’s microbiome module. We started by simulating a bistable memory element – or balanced toggle switch – based on the circuit initially developed by Gardner *et al.*^10^ due to the relative abundance of literature and characterization^29^,^30^. This circuit would potentially allow us to confirm that our system could capture the ‘toggle’ behavior of the microbiome circuity in the robot’s behavior, thus serving as a proof-of-concept for our proposed tool. The complete details of our analytical modeling and computational simulation approach is described in Supplementary Text S2.

**Figure 3:**
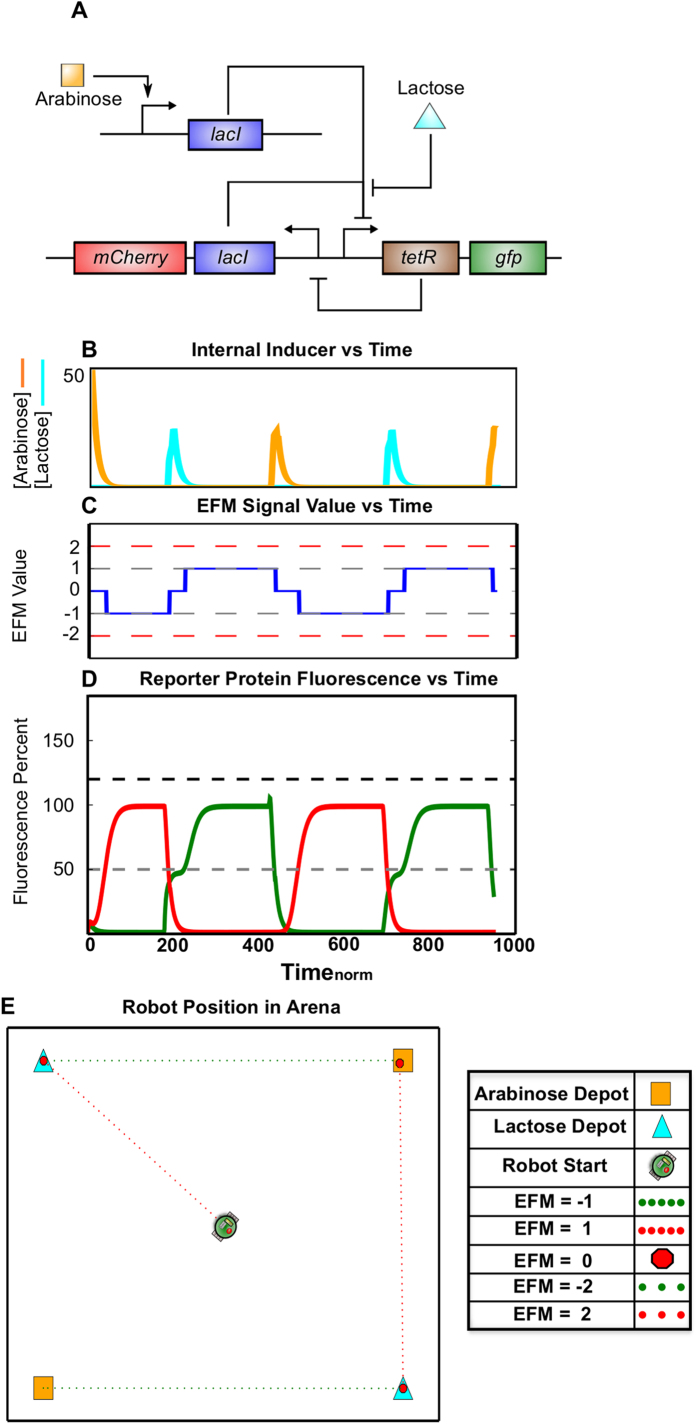
Emergent Robotic Host Behavior Resulting from a Microbiome with a Bistable Memory Circuit. (**A**) A bistable switch – or balanced genetic toggle switch – was simulated. The gene topology is represented using systems biology network notation. (**B**) Simulation results for internal inducer concentrations of lactose (cyan) and arabinose (orange) - see Supplementary Figure 4 for more details. (**C and D**) Simulation results for internal fluorescent protein reporter concentrations of mCherry (red) and GFP (green) are shown in (**D**). These are parsed into the EFM electronic output shown in (**C**). (**E**) A simulation of resulting robot motion depicts movement at constant velocity through the arena with stops (larger red octagons) to dock at individual carbon depots.

As expected, the results of these simulations (Fig. 3) show a robotic platform that alternates between seeking arabinose and lactose carbon depots. Upon initial activation with a transient pulse of an inducer, a balanced genetic toggle (Fig. 3A) drives sustained expression GFP or mCherry, and in the topology shown here, can be ‘flipped’ by the external addition of either lactose or arabinose. The resulting temporal, biochemical landscape (Fig. 3D) drives a spatiotemporal robot behavior (Fig. 3E) characterized by its bistability. Repeated simulations always showed that a balanced toggle switch caused the robot to seek out a balanced set of depots (i.e., two lactose and two arabinose) in our virtual environment.

In order to evaluate the robustness of this bistability, we simulated the balanced toggle and included stochasticity^31^,^32^ in the *in silico* system’s engineered living microbiome. These simulations (Figure S3) showed maintenance of the system’s bistable memory, accompanied by additional stalls along motion paths. We found the system’s behavior to be more sensitive to large degrees of stochasticity in translation in comparison to transcription. However, these large degrees of translational stochasticity are unlikely to occur in a physical, built system as most stochastic variability in prokaryotic protein synthesis has been previously shown to result from noise in transcription^33^,^34^ rather than translation.

Finally, we tested the robotic platform’s toggling behavior at an environmental level by simulating carbon depots that appeared randomly, rather than at fixed vertices (Figure S4). This simulation upheld the bistability of the robotic behavior, continuing to result in a robot that alternated its motion between lactose and arabinose sources regardless of the inducer carbon depot location.

### Simulation: Biased Toggle Switching in Engineered Microbiome and Robot Behavior

However, attaining a truly balanced toggle switch is difficult in the laboratory^35^, and often wet-lab molecular bioengineering results in an unbalanced, or biased, toggle switch. This circuit lacks the stable equilibrium seen in the balanced toggle switch^10^, with a tendency to transcribe and translate one side of the circuit even when no inducers are present. This imbalance is driven by many factors including promoter strength, ribosome binding site (RBS) strength, and protein and mRNA degradation rates. A biased toggle switch can provide timer-like behavior useful for cellular control of processes ranging from metabolism to apoptosis. In order to discover how this genetic feature in the commensal microbiome alters host behavior, we simulated a biased toggle with an imbalance between the RBS strengths controlling LacI and TetR translation (See Supplementary Text S3).

**Figure 4:**
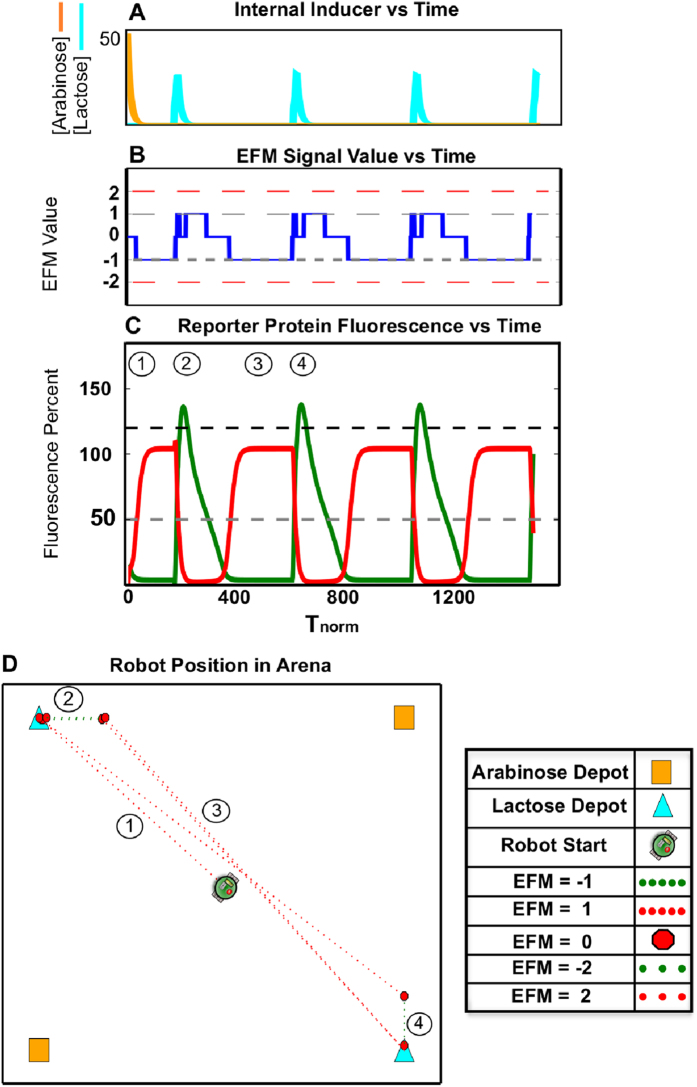
Emergent Robotic Host Behavior Resulting from a Microbiome with an Unstable Memory Circuit. A biased switch – or unbalanced genetic toggle switch - with the topology shown in Fig. 3A was created by increasing the ribosome binding site (RBS) for LacI to be 2.4 times the strength of the RBS for TetR. (**A**) Simulation results for internal inducer concentrations of lactose (cyan) and arabinose (orange) (**B and C**) Simulation results for internal fluorescent protein reporter concentrations of mCherry (red) and GFP (green) are shown in (C). These are parsed into the EFM electronic output shown in (B). (**D**) A simulation of resulting robot motion depicts the robot behaving in a manner different from Fig. 3E, with a clear preference for lactose carbon depots. Specifically, the robot briefly seeks arabinose depots after a lactose depot is acquired, however this period is quickly overwhelmed by the biased toggle switch behavior and the robot changes course to seek out a lactose depot.

The resulting robot path (Fig. 4D) suggests a behavioral ‘preference’ for lactose. This is attributed to the timer-like behavior of the genetic circuit demonstrated in the temporal reporter protein landscape (Fig. 4C), wherein a spike in GFP production caused from exposure to lactose depots is quickly attenuated by a genetic bias for LacI and thus, mCherry synthesis. This behavior is caused by a difference in the LacI and TetR RBS ratio creating a translational imbalance for the repression proteins.

### Simulation: Toggle Switch Parameter Sensitivity

**Figure 5:**
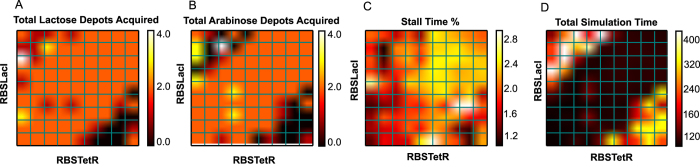
Exploration of Toggle Switch Parameter Space. This figure presents how changing RBS strengths driving LacI and TetR expression can change the robotic platform’s behavior without altering the genetic topology. (**A**) The total number of lactose depots acquired by the robot. (**B**) The total acquired arabinose depots acquired by the robot. (**C**) The percentage time the robotic platform spends in stall (i.e., EFM = 0). (**D**) The total time steps of the simulation. These figures demonstrate behavioral bifurcations driven exclusively by RBS strength.

The results shown in Fig. 4 also raise an important question: without altering the genetic topology, what microbiome biochemical parameter(s) impact the emergent behavior of the robotic host? In order to explore this question, we performed a parameter sweep for the same RBS_LacI_ and RBS_TetR_ used to simulate Fig. 4. By evaluating quantitative metrics for behavior, such as simulation runtime and depots acquired, we were able to capture shifts in the robot’s behavioral regime (Fig. 5) driven exclusively by RBS strengths and the resulting toggle bias.

This parameter sweep also provided evidence of behavioral bifurcations seen in the yellow region to the upper right of Fig. 5C and in the intense black band in Fig. 5D. Although not immediately apparent, these areas represent distinctly different emergent robotic behavior than surrounding regions. The yellow region (Fig. 5C) is characterized by a high percentage of time spent at zero velocity, which we defined as ‘stalling’, for the robot. The black regions (Fig. 5D) represent time efficient host behavior, with a minimum amount of time spent stalling with an EFM value = 0. In conjunction, these differences in parameter space suggest potential host performance optimization caused by microbiome physiology.

### Simulation: Host Feedback to the Engineered Microbiome

**Figure 6:**
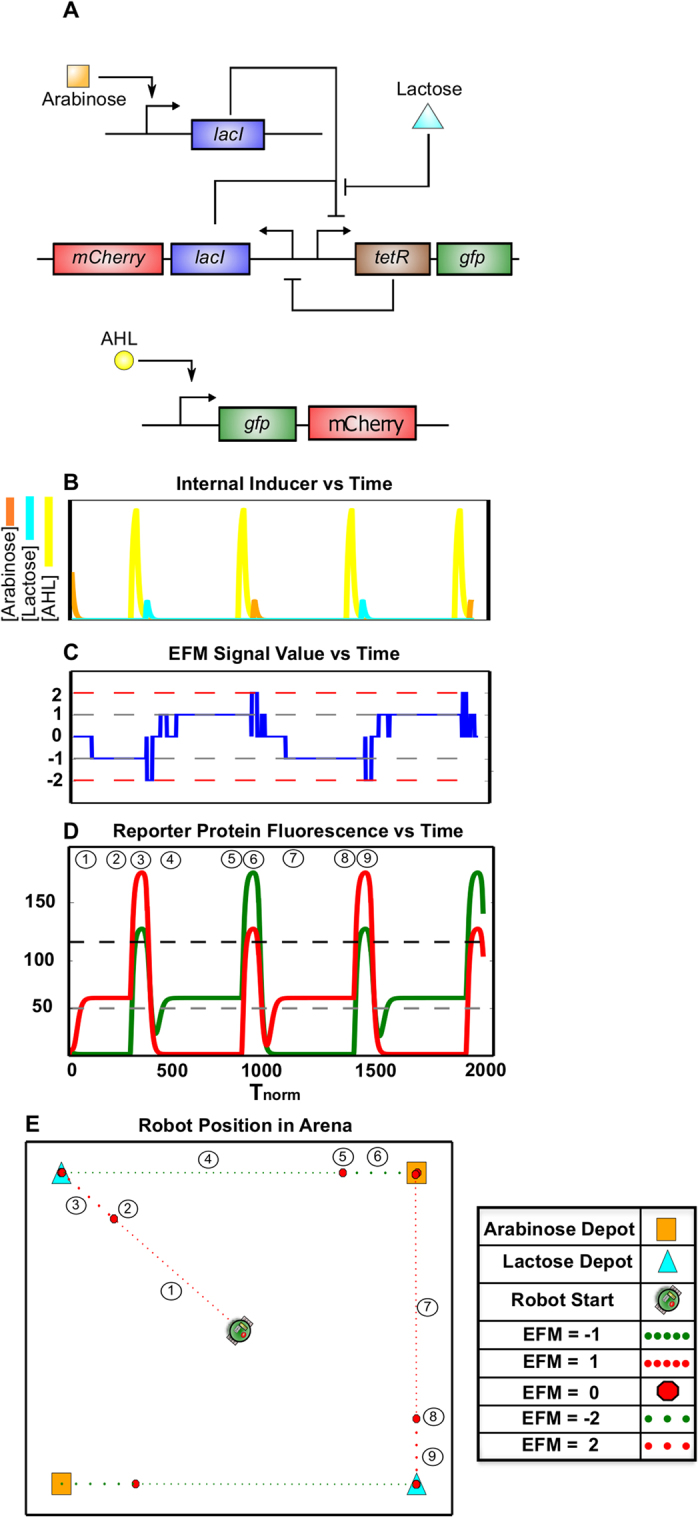
Addition of Orthogonal Operon Yields Nuanced Predation Habits. (**A**) The toggle switch topology modified with an additional, orthogonal operon containing the P_lux-λ_ promoter driving polycistronic expression of GFP and mCherry was simulated. This promoter is induced by AHL, which the robot is programmed to inject into the living, onboard microbiome when it nears any carbon depot. (**B**) Simulation results for internal inducer concentrations of lactose (cyan), arabinose (orange), and AHL (yellow). (**C and D**) Simulation results for internal fluorescent protein reporter concentrations of mCherry (red) and GFP (green) are shown in (**D**). These are parsed into the EFM electronic output shown in (**C**). Note the addition of EFM values of 2 and -2 indicating the robot is moving at two times the base velocity. (**E**) A simulation of resulting robot motion depicts the robot moving towards a depot, pausing, and then moving at twice the speed when close to the depot. This behavior appears to be qualitatively similar to stalk-pause-strike predation, an identifiable trait in higher level organisms.

Having demonstrated different host behavior regimes that result from defined parameter spaces, we modified the genetic topology to include feedback from the robotic platform. This created a robotic simulation that included two way communication between the microbiome and the robotic platform. The resulting robotic behavior was more nuanced than the toggle switch, and was analogous to predation habits found in nature. By adding an orthogonal operon consisting of a P_lux-λ_ promoter^36^ that simultaneously drives GFP and mCherry expression (Fig. 6A), we created a mechanism by which the microbiome can interpret an acyl-homoserine lactone (AHL) pulse delivered by the host robotic platform (i.e., through the execution of subroutine 6 shown in Supplementary Table 2). Thus, although we previously observed microbiome-to-host information flow, this additional circuitry allowed us to study host-to-microbiome feedback as well.

Figure 6 details the results from simulating this additional engineered gene circuit within the microbiome. The simulation shows an interesting nuance in robot behavior. Rather than seeking out carbon depots directly, the robotic platform pauses, and then travels at twice the previous, base velocity as it nears the depot. This behavior - reminiscent of predation - is a natural analogy to what is known as a stalk-pause-strike^37^ response in vertebrates.

### Simulation: Feedback Parameter Sensitivity

**Figure 7:**
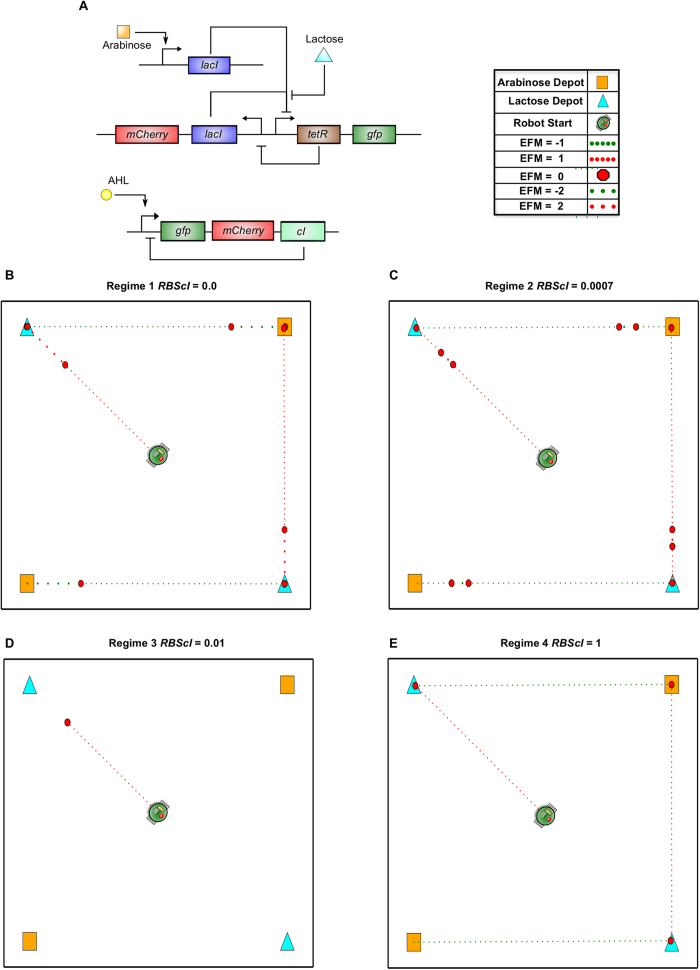
Distinct Behavioral Regimes Emerge from RBS Modification. (**A**) The gene circuit topology from Fig. 6A was further modified with an additional, orthogonal operon containing the P_lux-λ_ promoter driving polycistronic expression of GFP, mCherry, and critically, cI, the repressor from λ bacteriophage. In addition to being activated by AHL, this promoter is also repressed by cI, thus the new operon is auto-repressing. Furthermore, the robot is programmed to inject AHL into the living, onboard microbiome when it nears any carbon depot. (**B**) When the simulated RBS strength for cI (RBScI) is close to 0.0, the robotic platform behaves in the stalk-pause-strike manner described in Fig. 6.(**C**) With the RBScI value at 0.0007, there is a decrease in the length of the ‘strike’ period of the predation pattern leading to a stalk-pause-strike-pause-stalk behavioral regime. (**D**) Increasing the RBScI value to close to 0.01 leads to a regime of inactivity whereby the robotic platform is unable to acquire even one carbon depot. (**E**) Finally, as the RBScI value approaches 1, the system behaves similarly to the initial balanced toggle switch seen in Fig. 3. These multiple, and strikingly different, host behavioral regimes indicate how biochemical networks of the microbiome may have drastic impacts on host behavior.

Finally, we demonstrated tunability in the host-microbiome feedback by adding an additional *cI* gene driven by the P_lux-λ_ promoter (Fig. 7A) and modifying the corresponding RBS strength (RBScI). Our results show a number of distinct robotic behavioral regimes (Fig. 7) including both toggling and stalk-pause-strike behaviors previously noted.

The results from this RBScI parameter sweep capture four distinct robot behavioral regimes when varying RBScI from 0 to 1 (i.e., over the extremes of a relative range). When the RBScI value is close to zero (Fig. 7B), we observe the stalk-pause-strike behavior seen in Fig. 5. This regime is expected as a low RBScI value (<0.0007) would imply that a negligible amount of cI is translated. As we increase the RBScI value (0.0007–0.001), we observed a regime of stalk-pause-strike-pause-stalk previously unseen (Fig. 7C). Within this regime, the robot moves towards a depot (EFM ± 1) and then stops motion (EFM = 0). After a brief pause, it begins traveling at twice the base velocity (EFM ± 2) before pausing again (EFM = 0) and finally finishing its approach of the depot at base velocity (EFM ± 1). Further raising the RBScI value (0.001–0.8) caused the robotic host to enter a regime of permanent stall (Fig. 7D), wherein the robot acquires no carbon sources. Finally, as the RBScI value approaches 1 (Fig. 7E), the robot behaves in the same bistable manner seen in Fig. 3. This bistability is the result of quickly appearing, large [cI] that auto-represses its further transcription from the P_lux-λ_ promoter. These different regimes demonstrate our ability to tune robotic behavior by altering only a single genetic parameter within the microbiome.

## Discussion

Although interconnectivity between commensal bacterial physiology and host behavior has been experimentally observed^5^, the underlying biochemical interactions^38^ have yet to be fully understood. Here, we have created a unique *in silico* tool that enables us to explore this relationship with synthetic biology. Much in the way that synthetic gene circuits allows the exploration of genetic pathways and relationships in a single organism^39^, this tool could be used to augment and examine the interconnected networks that drive host-microbiome interactions.

Crucially, we explored two different topologies of information flow critical for host-microbiome interactions.

First, by simulating the toggle switch, we examined information flow from the environment to the microbiome, and then to the robotic platform. This system design (Fig. 3) allowed us to establish an initial behavior theme: host alternation between nutrient sources (i.e., lactose and arabinose carbon depots) resulting from a repeatedly toggled, bistable gene network. We then demonstrated that a translational parameter, RBS strength, could serve as a tunable component for modifying the robot’s affinity for these nutrient sources. Thus, we were able to use both genetic topology and parameter strength to prescribe a range of robot behaviors (Figs 4 and 5).

However, host-microbiome systems in nature are not limited solely to microbiome-to-host communication. They also include mechanisms for host-to-microbiome information flow^40^. By adding the additional P_lux-λ_ driven circuit and subroutine 6, we included this feature in our robotic system. In doing so, we simulated a system capable of mimicking host-microbiome interactions found in nature (Fig. 6). The addition of this circuit resulted in robot behavior analogous to stalk-pause-strike vertebrate predation^37^. Furthermore, performing a one-dimensional parameter walk (i.e., varying the RBS strength driving cI expression) within this genetic topology showed that multiple distinct robot behaviors could be modulated by this single parameter (Fig. 7). In addition to predation-like movement, these behaviors ranged from alternating between carbon source depots to permanently stalling. Our results demonstrate that small changes in biochemical parameters can result in the emergence of very different host robotic behaviors.

Our model system provides a useful system for exploring host-microbiome interactions with synthetic biology. By integrating an engineered microbiome, a microfluidic chemostat mimicking the microbiome’s environment within an organism, and a robotic conveyance, we have designed, modeled, and simulated a biomimetic system that allows us to explore natural phenomena through both synthetic biological and robotic programming. We expect this model system will have implications in fields ranging from synthetic biology and ecology to mobile robotics.

## Methods

All numerical simulations were programmed in MATLAB® 2014a using the Simulink™ software package. Our simulations relied upon a combination of continuous and discrete functions, interacting in a block-based model (see Supplementary Information for additional details). In order to facilitate accurate updating of state conditions, all integrations were calculated using MATLAB’s ode5 numerical method approach with a fixed time step. Ode5 is an implementation of the Dormand-Prince^41^ algorithm based off of Runge-Kutta approaches.

For every test cycle, all initial conditions were set at zero with the exception of internal arabinose concentration which had an initial condition of [50]. Each simulation ran through four iteration cycles corresponding to the four available carbon depots.

Data analysis and plotting of simulation results were performed using MATLAB and Python, respectively. Python libraries *numpy*, *scipy* and *matplotlib* were leveraged for the creation of graphics. Graphics were formatted as .SVG files and edited in InkScape® as vector images. Non-data graphics were created and edited exclusively in InkScape.

All Simulink and MATLAB files are made available upto request.

All simulations were run on an ASUS Zenbook UX32VD running with an Intel® Core™ i7-3517U processor at 1.90 GHZ and 2.40 GHZ with 10 GB of RAM on a 64-bit Windows 8.1 operating system. Average runtime for each simulation trial was 2.78 minutes for the basic toggle switch (Fig. 3) and incrementally more (<0.1 minute) for other circuits.

## Additional Information

**How to cite this article**: Heyde, K. C. and Ruder, W. C. Exploring Host-Microbiome Interactions using an *in Silico* Model of Biomimetic Robots and Engineered Living Cells. *Sci. Rep.* **5**, 11988; doi: 10.1038/srep11988 (2015).

## Supplementary Material

Supplementary Information

## Figures and Tables

**Figure 1:**
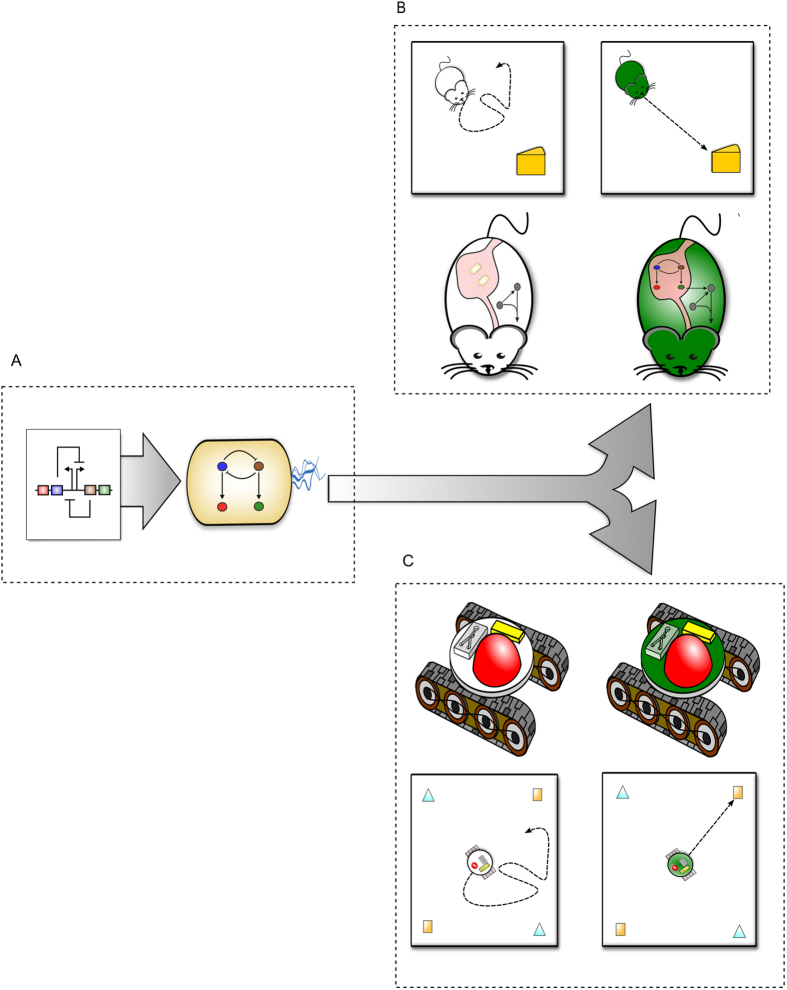
Living Cells Interfaced with a Biomimetic Robot as a Model System for Host-Microbiome Interactions. (**A**) A synthetic gene network – also known as an engineered gene circuit. Uploading a gene circuit into living bacteria endows cells with a programmable biomolecular network. (**B**) Engineered bacteria and their circuits can be introduced into an organism’s microbiome. The networks of the host and microbiome combine to form a complete gene network. In the absence of the complete host-microbiome network, host behavior is erratic. A programmed microbiome drives new, and potentially rational, host behavior. (**C**) A robot with a microfluidic chemostat mimics the microbiome’s environment within an organism. The robot is conceptualized to include a miniature fluorescent microscope, along with the pumps necessary to deliver inducers to the onboard microfluidic chemostat. This microscope allows for modulations in the reporter protein levels to be interpreted by the robot electronically. In the absence of a living microbiome, robotic host behavior can be erratic. A programmed, living microbiome drives new host robotic behavior.

**Figure 2:**
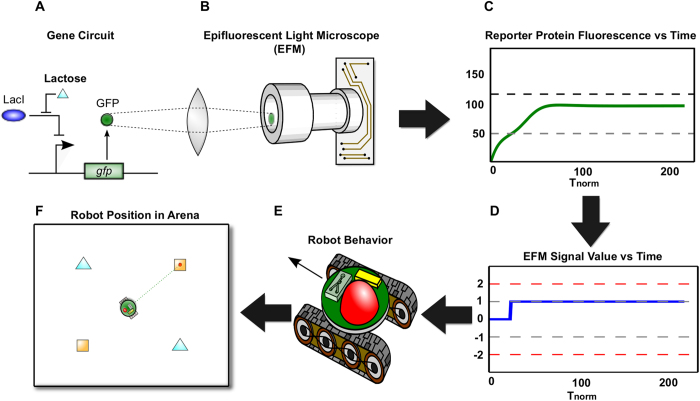
Computational Simulation Approach for the Model System. (**A**) A basic gene circuit – the *lac*-inducible gene network – forms the core of all simulated gene network behavior. (**B**) Green fluorescent protein (GFP, shown as a green dot) from this circuit is conceptualized to be detected by an onboard miniature, epifluorescent microscope (EFM). (**C**) A computational simulation of microbiome GFP production based upon an analytical model for the circuit in (A). In a built-system, this protein fluorescence signal would be the light detected by the EFM. (**D**) The conceptualized robot uses onboard electronics to convert the measured light signals into electrical (voltage) signals. (**E**) Voltage signals meeting specific criteria activate pre-programmed robot motion subroutines. (**F**) The resulting emergent behavior potentially leads a robot to a carbon fuel depot. Here, robot behavior resulting from a simulation of the circuit in (A) is shown. The robot was programmed with motion subroutines that activate to seek arabinose (orange square) depots following receipt of lactose (cyan triangles).
