# Supplementary material for: Exploring Host-Microbiome Interactions using an in Silico Model of Biomimetic Robots and Engineered Living Cells
Source: Sci Rep. 2015 Jul 16;5:11988. doi: 10.1038/srep11988 (PMC4648426; doi:10.1038/srep11988)
Supplement: Supplementary Information [file srep11988-s1.pdf]

SUPPLEMENTARY INFORMATION

Exploring Host-Microbiome Interactions using an *in Silico* Model of Biomimetic Robots and Engineered Living Cells

Keith C. Heyde[1] and Warren C. Ruder[,2]*

*Corresponding Author

[1]Department of Biomedical Engineering and Mechanics
[2]Department of Biological Systems Engineering
Virginia Polytechnic Institute and State University
Blacksburg, VA 24061

SUPPLEMENTARY FIGURES

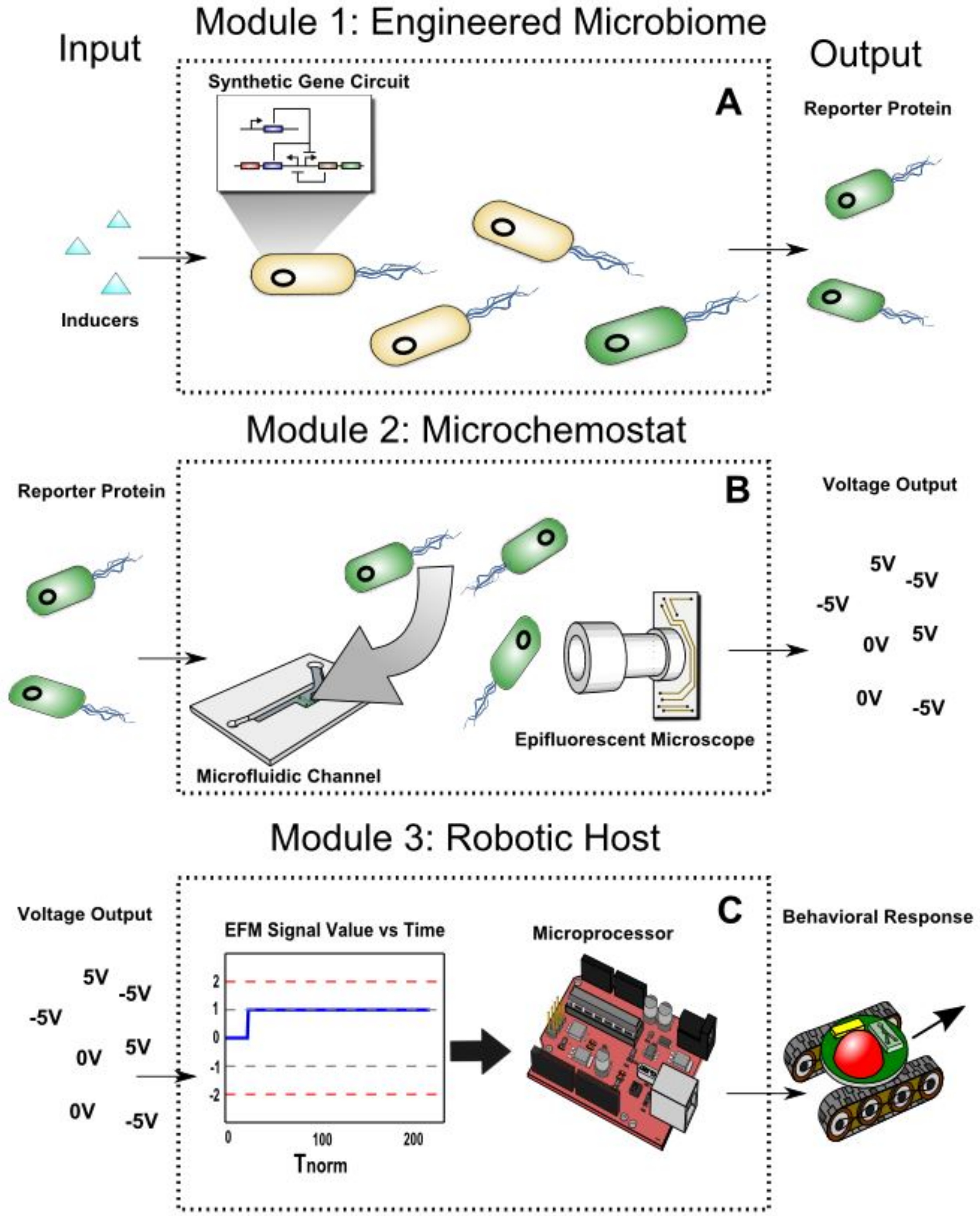

Figure S1. Robotic Platform Information Flow. This figure shows a visual representation of information flow through the three modules. (A) The synthetically engineered microbiome, programmed with a synthetic gene network. (B) The microchemostat environment with physical microfluidic channel and epifluorescent (EFM) microscope. (C) The robotic host translating EFM signal through a microprocessor into robotic behavioral response.

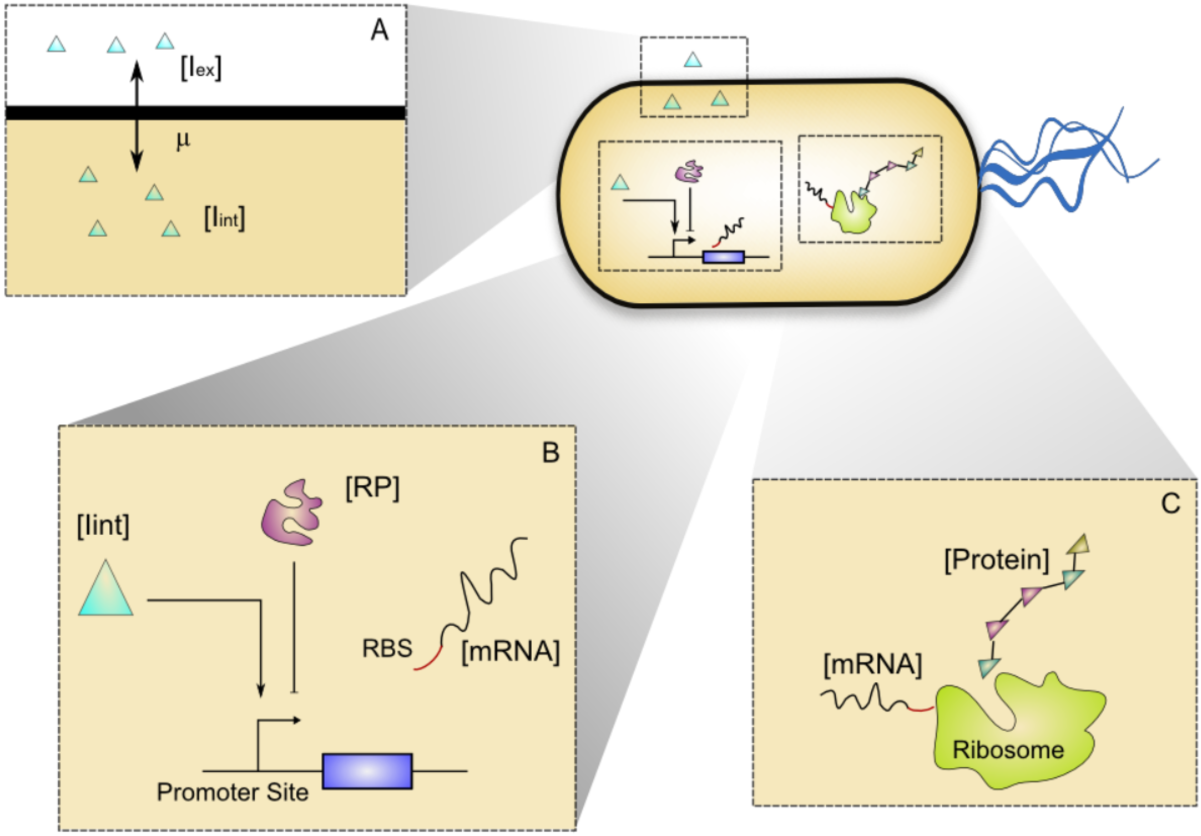

Figure S2. Biochemical Model Basics. This figure shows a visual representation of the biochemical model used for our simulation. (A) The inducer transport through the membrane barrier. (B) The interactions involved with the translation of [mRNA]. This includes internal inducers, a promoter site and repression proteins. An RBS is also seen as being associated with the mRNA. (C) An illustration of the translation event creating a protein, relating in [mRNA] to the [Protein] produced. This process is driven by a ribosome.

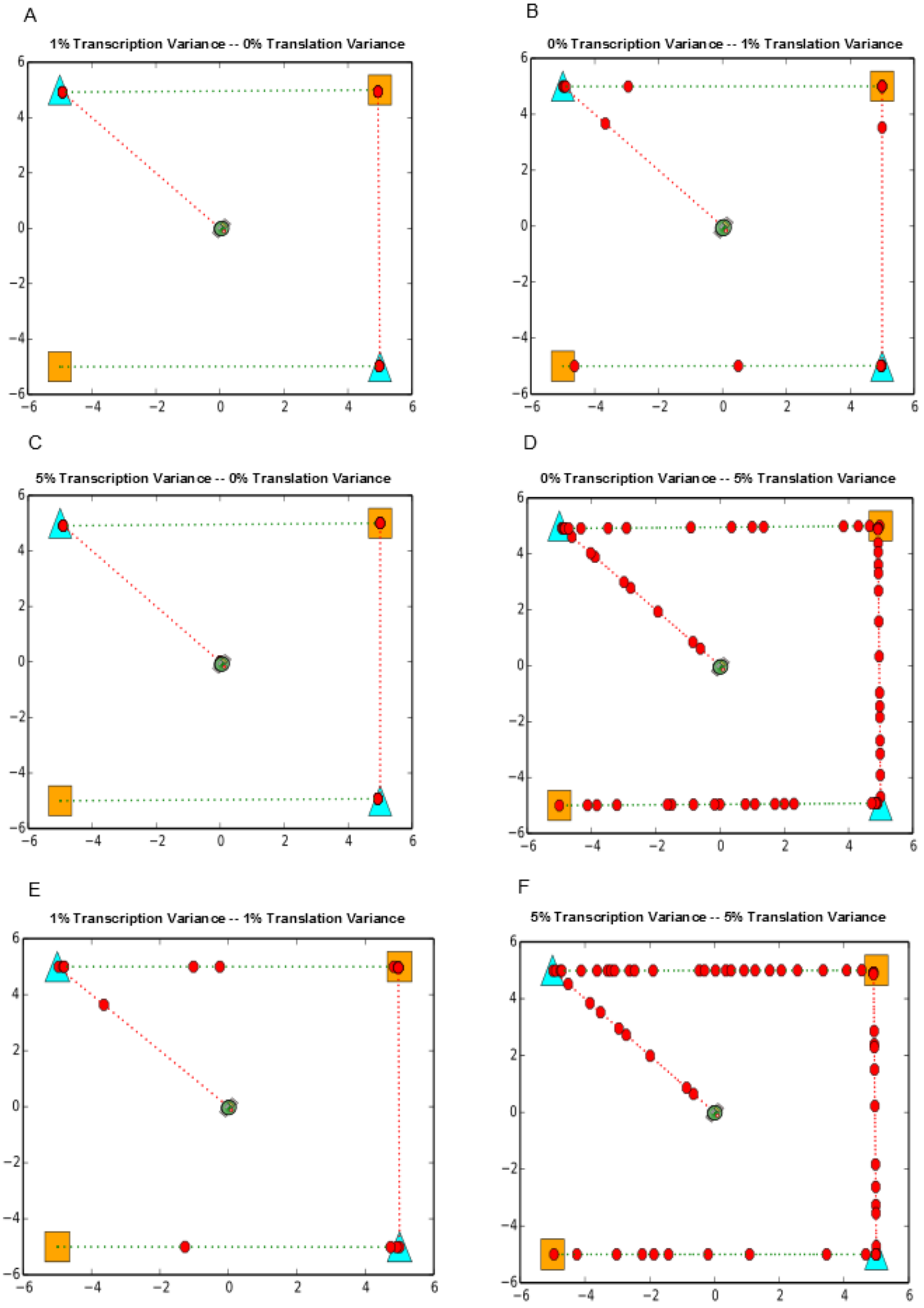

Figure S3. Exploring Stochasticity in the Simulated Gene Network. We ran six simulations exploring how stochasticity in transcription and translation affected the performance of the robot platform. The reporter protein, EFM signal, and inducer concentrations associated with panels A-G are presented in Figures S7-S12 respectively.

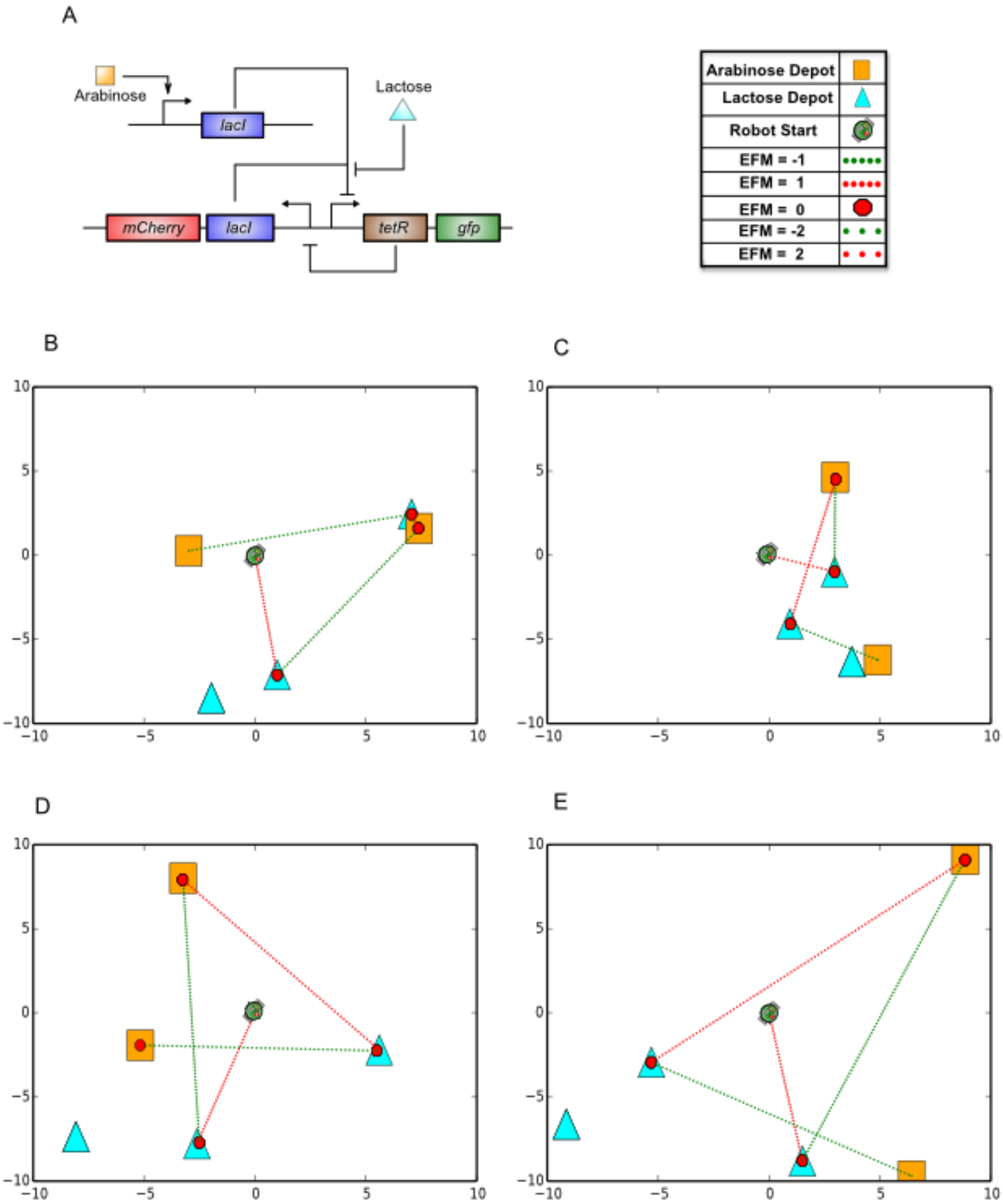

Figure S4. Balanced Toggle Switch with Randomly Occurring Carbon Depots. (A) The biomimetic robot host was endowed with bacterial cells containing a balanced toggle switch. (B-E) Four different simulations were run with randomly placed carbon depots. Each simulation showed the robot alternating between Lactose and Arabinose depots in a bistable manner.

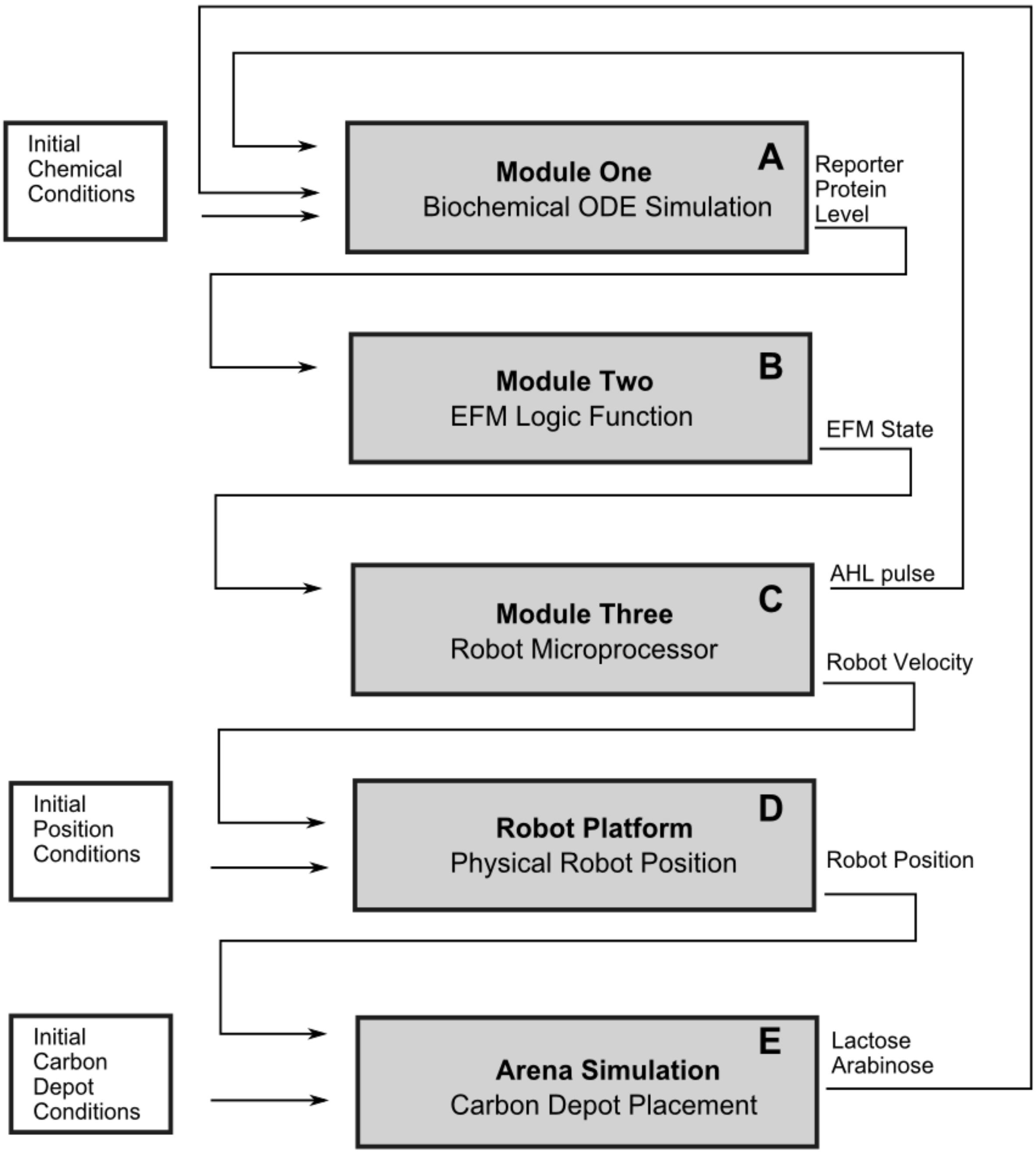

Figure S5. System Information Flow. We showed how variables passed between the five different simulation systems including the three modules from Figure S1 (A, B, and C), the robotic platform (D) and the arena simulation (E). Chemical, position, and voltage parameters are passed from systems, allowing for modularity of engineering.

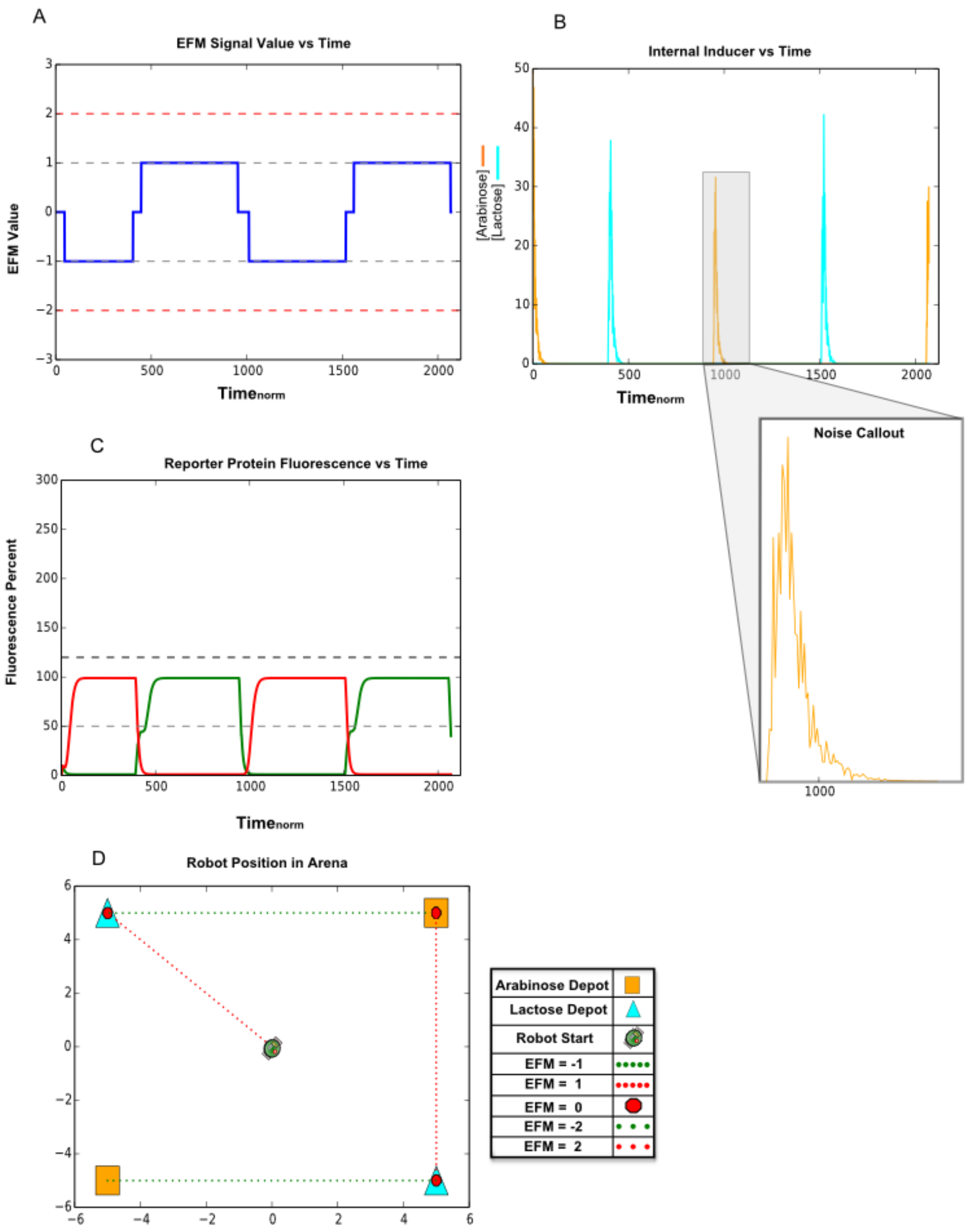

Figure S6. Stochasticity in the Internal Inducer Concentration. A Gaussian multiplier was applied to the lactose and arabinose internal concentrations. This multiplier had a variance that was 10% the previous time step's concentration. (A) The EFM signal for the stochastic circuit. (B) The lactose and arabinose internal inducer concentrations, with a callout box demonstrating the stochasticity of the signal. (C) The reporter protein levels. (D) The emergent robotic behavior within the arena.

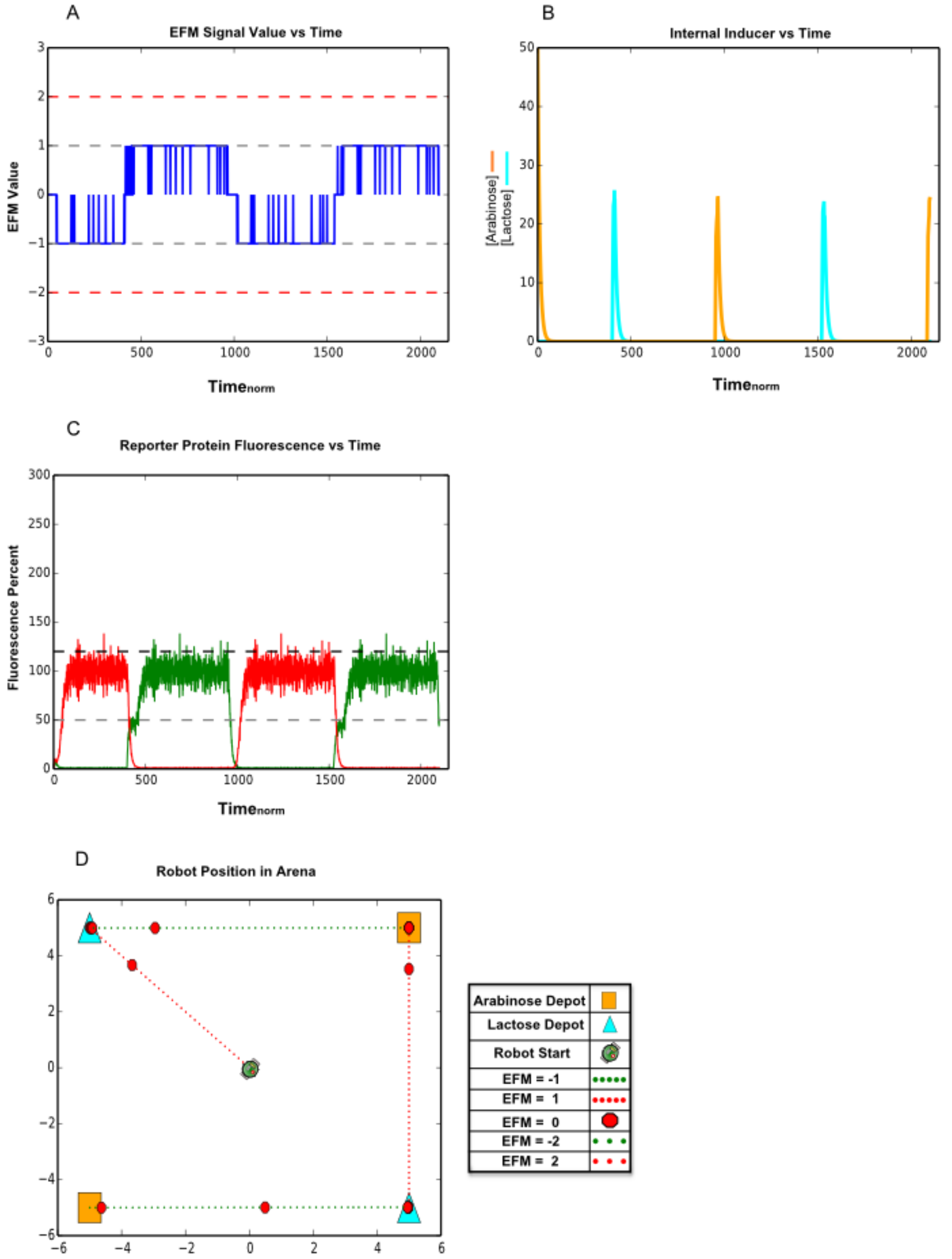

Figure S7. Stochasticity in a Balanced Toggle with 0% Transcription and 1% Translation variance. (A) The EFM signal for the stochastic circuit. (B) The lactose and arabinose internal inducer concentrations. (C) The reporter protein levels. (D) The emergent robotic behavior within the arena.

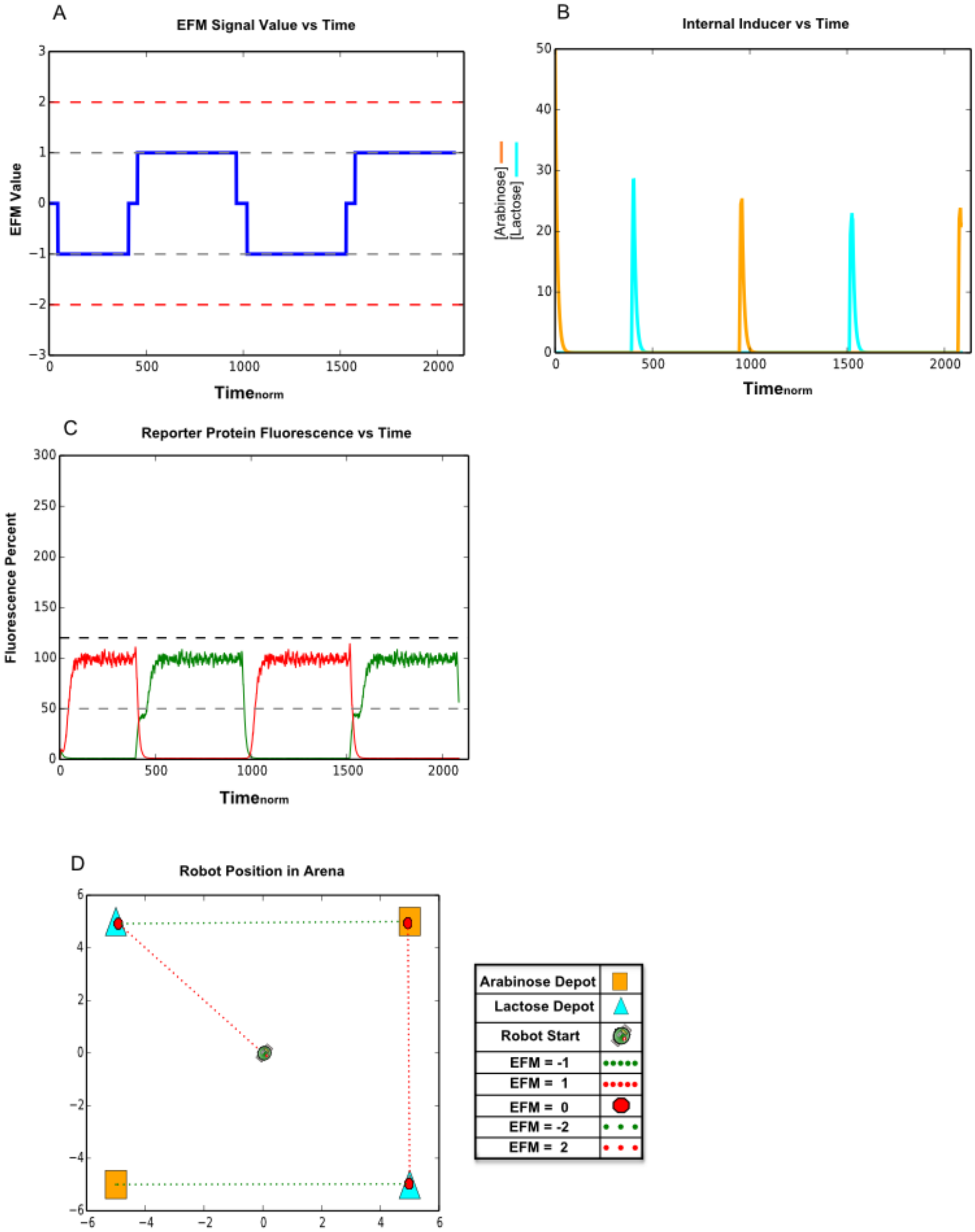

Figure S8. Stochasticity in a Balanced Toggle with 1% Transcription and 0% Translation variance. (A) The EFM signal for the stochastic circuit. (B) The lactose and arabinose internal inducer concentrations. (C) The reporter protein levels. (D) The emergent robotic behavior within the arena.

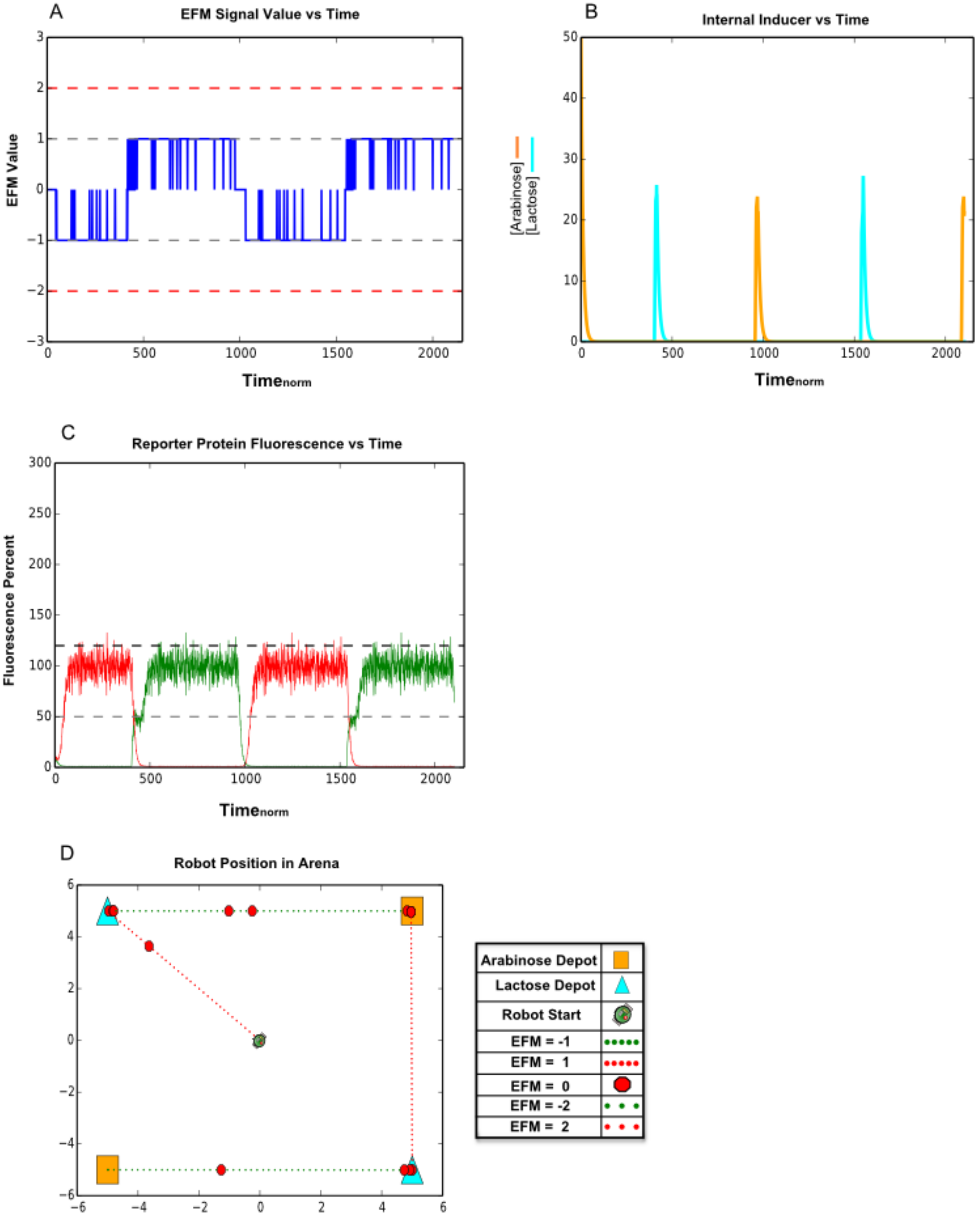

Figure S9. Stochasticity in a Balanced Toggle with 1% Transcription and 1% Translation variance. (A) The EFM signal for the stochastic circuit. (B) The lactose and arabinose internal inducer concentrations. (C) The reporter protein levels. (D) The emergent robotic behavior within the arena.

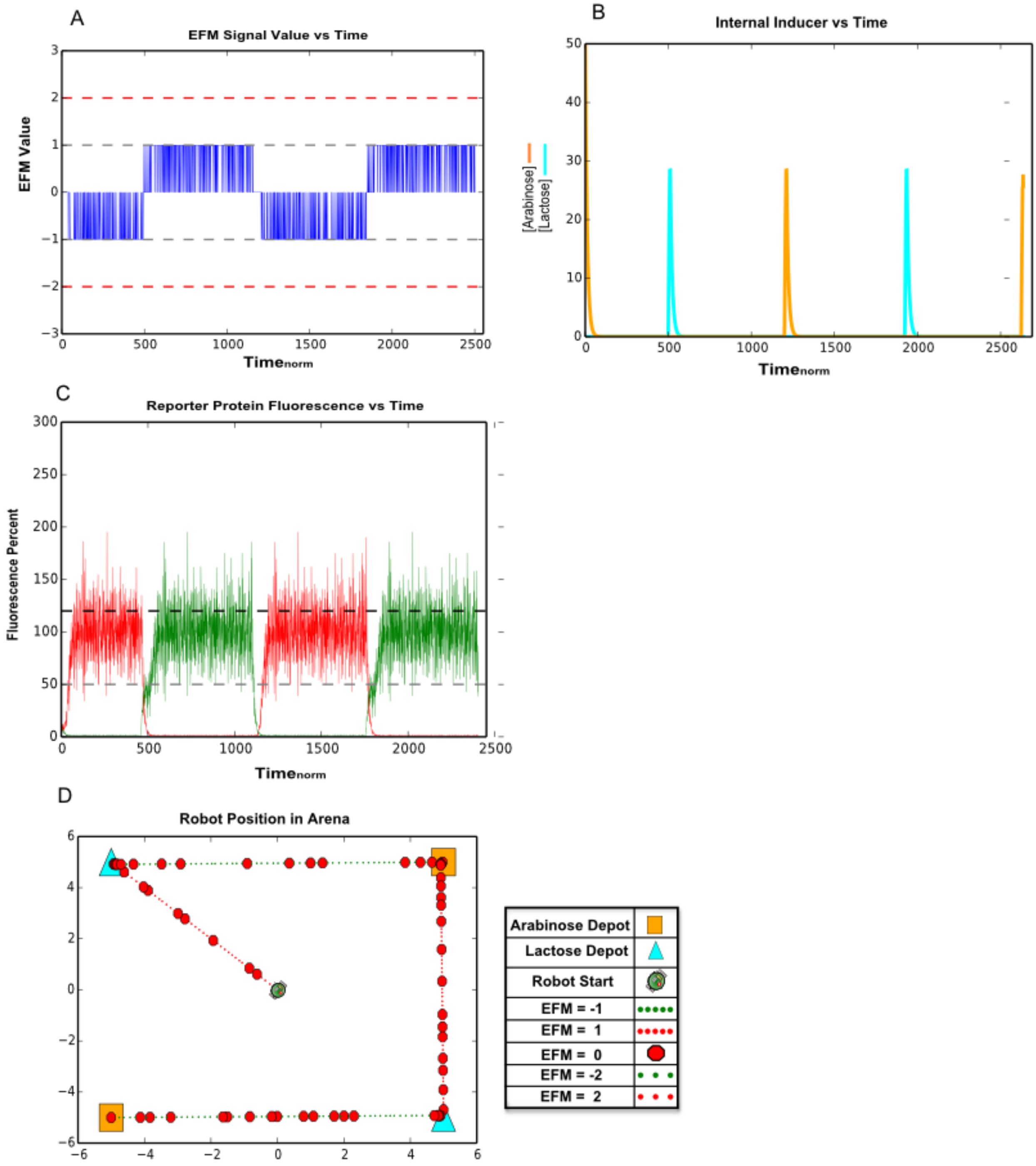

Figure S10. Stochasticity in a Balanced Toggle with 0% Transcription and 5% Translation variance. (A) The EFM signal for the stochastic circuit. (B) The lactose and arabinose internal inducer concentrations. (C) The reporter protein levels. (D) The emergent robotic behavior within the arena.

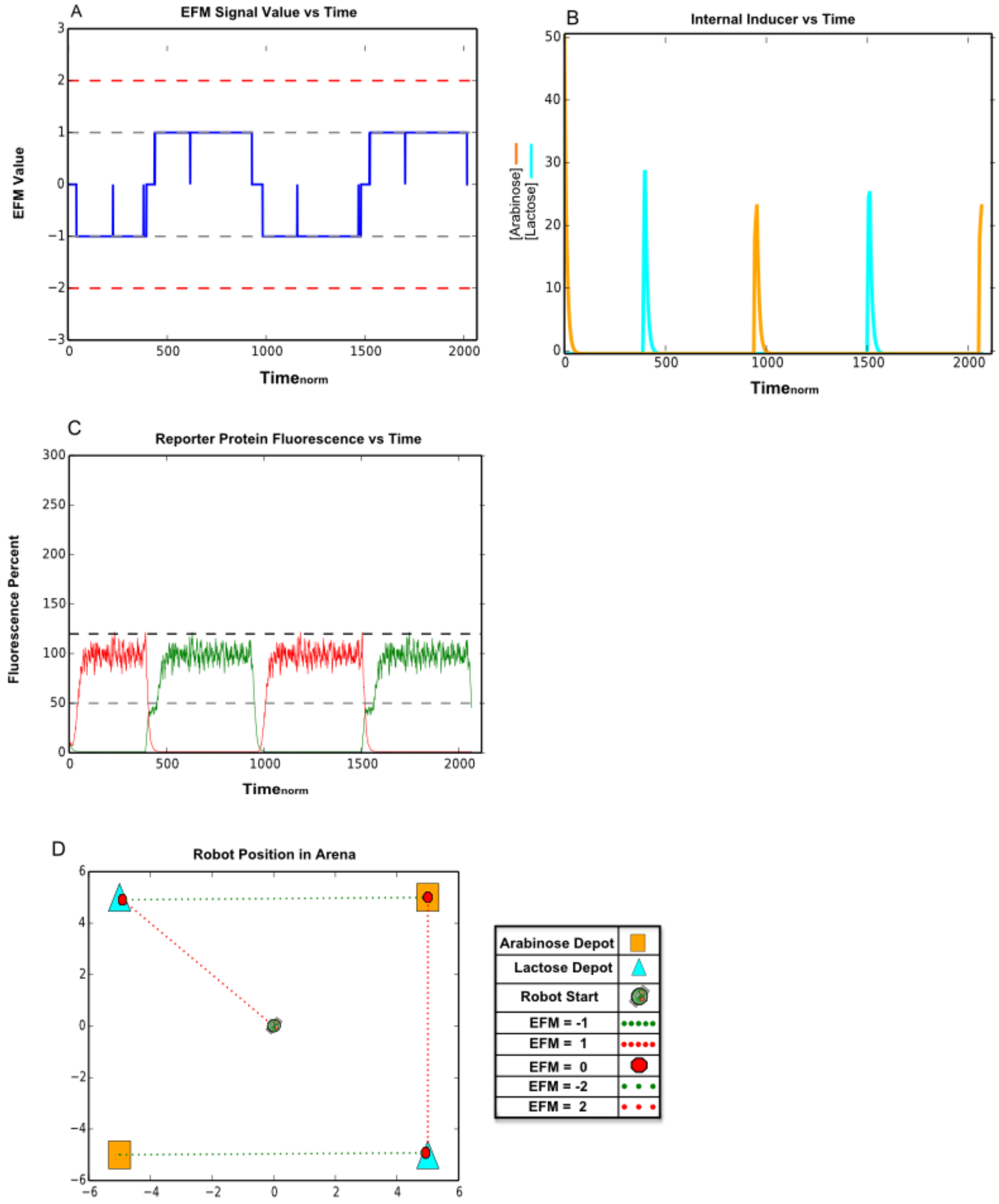

Figure S11. Stochasticity in a Balanced Toggle with 5% Transcription and 0% Translation variance. (A) The EFM signal for the stochastic circuit. (B) The lactose and arabinose internal inducer concentrations. (C) The reporter protein levels. (D) The emergent robotic behavior within the arena.

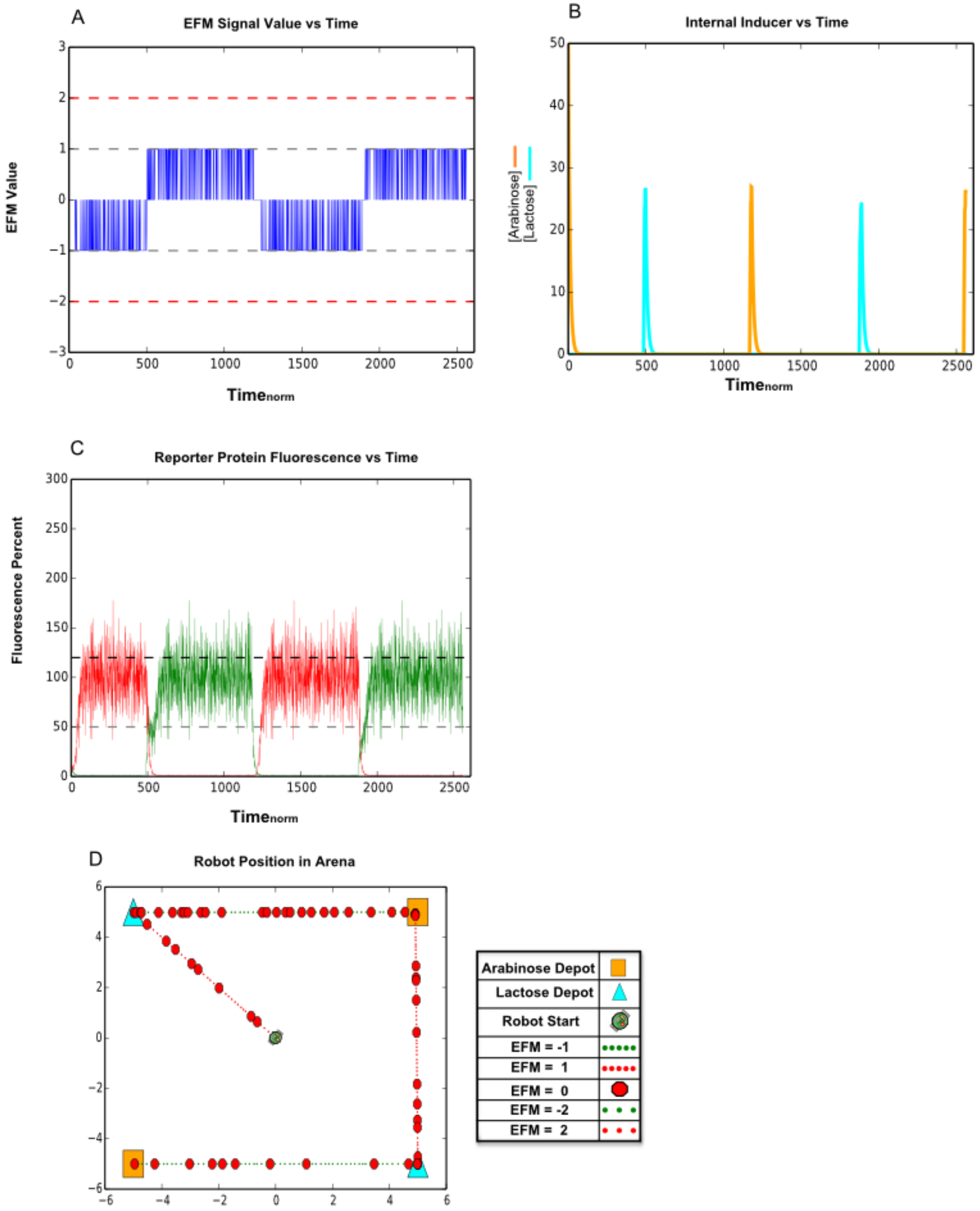

Figure S12. Stochasticity in a Balanced Toggle with 5% Transcription and 5% Translation variance. (A) The EFM signal for the stochastic circuit. (B) The lactose and arabinose internal inducer concentrations. (C) The reporter protein levels. (D) The emergent robotic behavior within the arena.

Supplementary Text

Text S1. Robotic System Design

Module One: Microbiome

Module one describes a living, engineered microbiome. Microbiomes in nature are an amalgamation of numerous species[1-3]. However we used a homogeneous, engineered *E. coli* population as a model system. This simplification is an established precedent[4,5] and allows us to experiment and engineer with a well-understood model organism before adding layers of complexity.

We chose to simulate the effects of synthetic gene circuits within the microbiome (Figure S2) by using a deterministic approximation common in the synthetic biology[6-9]. This framework centers on modeling the central dogma of molecular biology as a system of ordinary differential equations (ODE's) relating the rates of change of inducers, mRNAs, and proteins. Although this approach neglects the stochastic variation found in nature[10-14], it provides a simplified, modular, and computationally efficient framework for understanding gene network dynamics. We considered this simplification appropriate for our research due to the homogeneous nature of the bacterial population[15]. Nevertheless, we also have examined the role of stochasticity in our proposed system. We created several simulations accounting for transcriptional and translational noise[16,17] that are described later and shown in Figures S3.

The three primary equations for our ODE framework are shown below. An expanded derivation of these equations is presented in section Text S2 of the Supplementary Information.

$$\frac{d([[I_{int}])}{dt} = \mu([I_{ex}] - [I_{int}]) \quad \text{(S1)}$$

$$\frac{d([mRNA])}{dt} = \left(\frac{\alpha}{1+[RP]^{H}}\right) + \alpha_{Leak} + \left(\frac{k\times[I_{int}]}{1+[I_{int}]}\right) - HL_{mRNA}\times[mRNA] \quad \text{(S2)}$$

$$\frac{d([P])}{dt} = (RBS \times [mRNA] - HL_{ratio}\times[P]) \quad \text{(S3)}$$

Equation S1 is derived from analyzing the rate of change of the inducers inside of the cell. In the model described here, we simplified the endogenous network by envisioning a cell that had been engineered to no longer possess the native genes encoding arabinose and lactose metabolism; thus, the only incentive for seeking these inducers would be created by our engineered gene circuits. Therefore, we approximated the rate of change of the internal inducer concentration as a function of its transport across the cell membrane; this transport is governed by the gradient between the internal and external inducers concentration, $[I_{int}]$ and $[I_{ex}]$, respectively, and membrane permeability represented by a transport coefficient μ. In our model, inducers such as lactose and arabinose were introduced from the environment (Figure 3B, 4B, and 6B), or directly injected from the host robot (Figure 6A and 7A). The transport processes leading to our first-principal derived model for inducer concentration is shown in Supplementary Figure S2. Additional methods for modeling inducer concentration are noted in Supplementary Text S2.

As noted, our model assumes a homogenous, well-mixed bacteria population, kept in exponential phase. These assumptions allow us to describe the transcriptional and translational processes of synthetically engineered gene circuits as a mean population value, smoothing the fluctuations found at the single cell level[18]. Equation S2 incorporates these assumptions to describe the rate of change of mRNA. Leveraging existing deterministic models[8,19], we modeled temporal dynamics of mRNA as the sum of four terms. Under this paradigm, the first three terms on the right hand side of equation S2 relate the behavior of an inducible operon. Within these terms, [RP] is the concentration of a repressor protein with a corresponding Hill coefficient of H. α is a combined parameter describing a number of biophysical properties of the promoter site including transcription factor and RNAP binding affinity[8,19-21]. α_{Leak} is the rate of transcriptional leak of mRNA produced when the promoter site is repressed[8,19]. The inducer coefficient, k, is a parameter describing the rate at which mRNA is produced in proportion to the internal inducer concentration, $[I_{int}]$. Finally, the fourth term in the model describes kinetic rate of decay for the mRNA. Here we assumed a first order decay processes for the mRNA, represented by the HL_{mRNA} term. This transcription process is shown in Supplementary Figure S2.

Equation S3 describes the rate-of-change of protein within the cell. Specifically, this equation relates rate of protein produced with the concentration of mRNA within the cell. Fundamental to this model is the assumption that all mRNA transcribed can be translated. This assumption allows us to ignore mRNA inhibitors[22] and riboregulators[23]. Additionally, we are interested in incorporating (ribosome binding site) RBS strength as a tunable parameter for altering system behavior, an approach often used in synthetic biology[24-28]. Therefore, we included a relative RBS strength term within equation S3. The protein's rate of decay is approximated as first order, but of a different magnitude than that of the mRNA; this decay ratio is described by the parameter HL_{ratio}. This translation process is shown in Figure S2.

Module Two: Microfluidic Chemostat

Our design for an onboard, programmable microbiome leverages previous work in microfluidic based synthetic biology[29-32] to approximate host-microbiome feedback found in nature. We can conceptualize this module as containing two features: 1) the physical chemostat (Figures 1H and Supplementary Figure S1B) and 2) the miniaturized epifluorescent microscope (Figures 2B, and Supplementary Figure S1B).

The conceived chemostat (Figure 1H) is based on existing designs[30,32] and combines a microfluidic channel housing the first module with peristaltic pumps. By presuming that we constitutively pump a carbon source, such as xylose, through the chemostat, we can assume the cells remain both well-mixed and in exponential phase[30]. Additionally, we conceptualized our microfluidic chip as a part of a system permitting chemical injections from either the robot or the environment, allowing us to simulate a biomimetic proxy for information exchange with the microbiome.

The second module also contains a miniaturized epifluorescent (EFM) microscope based on previous designs[33]. This module provides the crucial interface translating phenotypic variations, in the form of reporter protein (mCherry and GFP) production (Figures 2B, 2C, 2D, and S1B), into electronic information encoded as voltage differentials. In this manner, the EFM serves as a crucial biotic-abiotic interface.

In order to convert these reporter protein fluorescent measurements into a useful digital signal, we designed a computable response function (Table 1) to interpret the reporter protein intensities and to translate the measurement into one of five discrete outputs. This logic function generates what we term the EFM value, a signal sent to the robotic host microprocessor. We set these EFM signal value thresholds to enable responses to distinct regimes of reporter protein concentrations outputted by our simulated model. By extension, in a physical system, these values would be based on fluorescent intensity recorded by the epifluorescent microscope.

Supplementary Table 1. EFM Signal Logic

Conditions	EFM value
[GFP] < [mCherry] AND [mCherry] > 120 AND [GFP] >120	-2
[GFP] < [mCherry] AND 50 < [mCherry] < 120 AND [GFP] < 50	-1
All Other Conditions	0
[GFP] > [mCherry] AND 50 < [GFP] < 120 AND [mCherry] < 50	1
[GFP] > [mCherry] AND [mCherry] > 120 AND [GFP] >120	2

Module Three: Robotic Mobile Prosthetic and Microprocessor

The third module is a robotic host and a microprocessor that controls all mechatronic behavior for the robotic platform. In our simulation, we designed the robot to have mobile functionality similar to the e-puck swarm robot[34]. Therefore, our robot is a tank robot with two-wheel actuation[35]. We conceptualized the robot as having the capability of seeking and docking with different inducer (lactose or arabinose) carbon depots in its environment.

In this proposed seek-and-dock process, the conceptualized robot would be endowed with transmitters and sensors enabling it to emit a wireless ‘ping’ signal to nearby carbon depots. These carbon depots would respond with a wireless signal with their location, and orientation so that the mobile robot could travel to them directly and ‘dock’ with the station using the correct angle-of-approach. The automated depot would then replenish onboard inducer concentrations with the docked robot.

The robotic host also includes hardware that allows it to ‘dock’ with an inducer carbon depot. This hardware would establish a watertight connection between the mobile robotic platform and the carbon depot. Once this seal has been established, the docking port would allow for the inducer to enter the microchemostat at a constant flow rate. This would provide an extracellular inducer concentration that would then enter the cells through membrane transport processes described in subsequent sections. During this docking, the robotic platform is still sensitive to the signals sent from the EFM.

Furthermore, we programmed the robot with a minimal set of subroutines (Supplementary Table 2) designed to mimic an organism’s mobile pursuit of nutrients (e.g., hunting) within its environment (Figure 1). These commands were programmed into an onboard

microprocessor that reevaluates subroutines states at every time step of the simulation (Supplementary Figure S1). This minimal set of subroutines allowed us to observe how the phenotypic state of the microbiome influences host behavioral response.

Supplementary Table 2. Programmed Robot Subroutines

Condition	Subroutine
EFM = -2	1: Locate, turn, and travel towards arabinose source at 2x base speed
EFM = -1	2: Locate, turn, and travel towards arabinose source at 1x base speed
EFM = 0	3: Do not move
EFM = 1	4: Locate, turn, and travel towards lactose source at 1x base speed
EFM = 2	5: Locate, turn, and travel towards lactose source at 2x base speed
Robot between 2 and 2.25 of carbon depot	6: Inject AHL pulse into onboard microfluidic channel.

The first five subroutines relate the robot's motion within the simulation environment. The last subroutine injects a pulse of a third inducer into the microchemostat directly from the robotic platform. This subroutine, sixth in Supplementary Table 2, is a simplification of the host-to-microbiome biochemical communication interaction found in nature. By including this biomimetic feature, we were able to create information exchange from the host to the microbiome (Figure S5), in addition to the other subroutines that enabled microbiome information to be passed to the host. We chose to mimic and simulate this biochemical communication using N-Acyl homoserine lactone (AHL) as the inducer molecule along with an AHL-sensitive, engineered promoter, $P_{lux\text{-}\lambda}$, due to this system's orthogonally and well characterized behavior[8,28,36-38].

Text S2. Model Derivation

Robot System Design

Our results are based on our design for a biomimetic robotic platform, engineered to simplify the host-microbiome interactions found in nature. This system allows us to capture five crucial information flows: environment-to-host (external sensors), environment-to-microbiome

(arabinose and lactose), host-to-microbiome (AHL pulse), microbiome-to-host (epifluorescent signal output) and host-to-environment (robot position). This information flow is presented in Figure 1 of the main text and Figure S1.

Biochemical Model Development

The biochemical simulation used in our model links inducer, mRNA, and protein concentrations. The field of computational molecular biophysics is vast, with established models existing for different scales and system complexity. One of the nuances when modeling cellular behavior is to select a modeling approach that captures the critical behavior while providing computational efficiency. For instance, one would not use a molecular dynamics model to explain the human circulatory system.

For our study, we needed to model reporter protein expression resulting from inducer concentrations that activate synthetically inserted genetic topologies. Within this context, we needed to relate synthetically programmable parameters, such as promoter and RBS strengths, in order to test our hypothesis. Fortunately, there is no shortage of existing models for understanding the transcription and translation processes. For example, the rise of systems and synthetic biology in conjunction with biophysics and numerical methods has opened the door for Monte Carlo/Markov Chain (MMMC) models used for even complex genomic networks[11,12].

However, although an understanding of the stochastic nature of gene expression is important, the design of our system allows us to use a continuous, deterministic approximation. This is a fair assumption given that our system focuses on macroscopic characteristics (reporter protein fluorescence) at a population level for a homogeneous culture.

Using a continuous framework, our first task was to describe the inducer concentration within the cell. From an inducer specific mass balance, we can describe change in inducer concentration as the sum of the transport of the inducer across the cell membrane and the degradation of the inducer by cellular kinetics.

$$\frac{\mathrm{d(Inducer)}}{\mathrm{dt}} = \text{Inducer Transport Into Cell} - \text{Inducer Metabolism} \qquad \text{(S4)}$$

Further exploring the terms on the right hand side, we can describe inducer transport into the cell by modifying Fick's law for diffusion over a transport barrier, given in equation S5.

$$\mathrm{J} = -\mathrm{D}\frac{\mathrm{dc}}{\mathrm{dt}} \qquad \text{(S5)}$$

In this equation, J is the diffusion flux measured in units of concentration per unit area per unit time, dc/dt is the inducer concentration gradient, and D is a transport diffusion coefficient that describes the ability for substances to flow through the membrane.

Our reactor is designed to be well mixed, and therefore we may assume dc/dt to be equal at all locations across the cell membrane for a given moment in time. This assumption allows us to assume equation S3 is true.

$$\frac{dc}{dt} = [I_{ex}] - [I_{int}] \quad (S6)$$

Whereby $[I_{ex}]$ is the inducer concentration external to the cell and $[I_{int}]$ is the inducer concentration within the cell membrane.

Additionally, within cells, D would normally behave as a function of membrane channel proteins such as AraFGH and permease[39,40]. However, for model simplification, we assumed these membrane proteins were held at a constant concentration, and therefore D was a constant. By additionally assuming all cells in our microbiome have a constant surface area, we can modify Fick's law by our assumptions to yield.

$$\text{Inducer Transport Into Cell} = \mu([I_{ex}] - [I_{int}]) \quad (S7)$$

Within equation S7, μ is a combined transport coefficient, defined as $\mu = D \times A$, with A being the cell's average surface area.

The second term on the right side of equation S4, inducer metabolism, requires a kinetic model to characterize the degradation of the inducer once inside the cell membrane. However, to simplify metabolism kinetics, we assumed that our engineered *E. coli* would include gene knockouts that eliminate the metabolism of the inducers used in our system; lactose, arabinose, and AHL. Therefore, the internal concentration of these three inducers may be characterized by equation S5.

$$\frac{d([[I_{int}])}{dt} = \mu([I_{ex}] - [I_{int}]) \quad (S8)$$

It should be noted that for all three of these inducers, $[I_{ex}]$ are controlled by non-cellular factors such as proximity to carbon depots and robot executable 6. In this way, the inducers serve as external signals linking information flow from the environment or robotic platform to the microbiome. We note that equation S8 represents first-order kinetics resulting in exponential decay of inducer concentration.

For the next variable, mRNA, we first set up a mass balance, noting that the rate of change for mRNA will equal the transcription rate minus the degradation rate. This equation S9 is formulated similarly to S4:

$$\frac{d(\text{mRNA})}{dt} = \text{rate of transcription} - \text{rate of mRNA degradation} \quad (S9)$$

In order to develop a term for the rate of transcription, we turned to existing literature for model development. However, we desired to make a modular, continuous approximation of operon behavior and therefore did not want to engage in promoter-site specific Shea-Acker's formalism[41]. Fortunately, existing work in synthetic genetic network dynamics[7,8,15] has developed a simplified continuous ODE for approximating inducible and repressible operon for behavior.

$$\text{rate of transcription} = \left(\frac{\alpha}{1+[RP]^H}\right) + \alpha_{Leak} + \left(\frac{k \times [I_{int}]}{1+[I_{int}]}\right) \quad (S10)$$

This equation relates the rate of change in mRNA production to a number of inputs driven by transcription events and mRNA degradation.

The first term on the right hand side is used to describe how the concentration of repression proteins [RP], such as TetR or LacI, affect the normal promoter-driven gene expression. Within this term, α is a coefficient describing the maximum transcription rate when no repression proteins are present. Finally, H is a term known as the Hill coefficient, and is used to describe the relative impact of a repression protein on an associated promoter.

The second term, α_{Leak}, is a term describing the 'leak' of a promoter. This term varies in accordance to the promoter studied. However, in our simulation we kept α_{Leak} to be roughly 1/100 of the α value.

The final term provides a mechanism for induced operon activation. Within this term, k is a signal coefficient that relates the amount of inducer to the rate of transcription.

Finally, for the rate of mRNA degradation, we developed a simple kinetic model relating the degradation rate to the half-life and concentration of the mRNA.

$$\text{rate of mRNA degradation} = -HL_{mRNA} \times [mRNA] \quad \text{(S11)}$$

It should be noted that many biological factors can alter the mRNA half-life such as nuclease tags. Furthermore, an mRNA's translational efficacy may be altered by the presence of riboregulators and other inhibitors. However, assuming these factors are not present, we may combine equations S9, S10, and S11.

$$\frac{d(mRNA)}{dt} = \left(\frac{\alpha}{1+[RP]^H}\right) + \alpha_{Leak} + \left(\frac{k \times [I_{int}]}{1+[I_{int}]}\right) - HL_{mRNA} \times [mRNA] \quad \text{(S12)}$$

Using a similar, but simpler model for protein translation and degradation, we are able to model the rate of change for protein as equation S10.

$$\frac{d([P])}{dt} = (RBS \times [mRNA] - HL_{ratio} \times [P]) \quad \text{(S13)}$$

Where [P] is the protein concentration, RBS is the ribosome binding site strength associated with the mRNA strand, and HL_{ratio} is the half-life ration of the mRNA to protein half-lives.

With S8, S12, and S13, we are able to write a set of governing biochemical equations shown in Supplementary Text Section 1.

Governing Biochemical Equations

$$\frac{d([[I_{int}])}{dt} = \mu([I_{ex}] - [I_{int}]) \quad \text{(S1)}$$

$$\frac{d([mRNA])}{dt} = \left(\frac{\alpha}{1+[RP]^H}\right) + \alpha_{Leak} + \left(\frac{k \times [I_{int}]}{1+[I_{int}]}\right) - HL_{mRNA} \times [mRNA] \quad \text{(S2)}$$

$$\frac{d([P])}{dt} = (RBS \times [mRNA] - HL_{ratio} \times [P]) \quad \text{(S3)}$$

These governing equations are also shown in Figure S2

Text S3. Simulation Design

In order to simulate the interplay between host, microbiome, and environment, we designed a block system architecture that combined continuous biochemical readouts with finite-state-machine logic functions for the epifluorescent microscope and microprocessor state. The general concept for this design is presented below in Figure S5.

Within our simulation, we used a fixed time step evaluation of state variables (protein, mRNA, inducer, position) to determine which subroutine the robotic platform should run. With this fixed time step evaluation in mind, we intentionally selected a fixed time step numerical method to evaluate the biochemical system. In this way we were able to best represent the engineering constraints of our designed biomimetic robot.

All of our Simulink and MATLAB files detailing the model parameters are available by request from the authors.

Balanced and Biased Toggle Switch

Here, we have including the set of governing ODEs for the genetic toggle switch presented in the paper:

i. $\frac{d([\mathrm{Arabinose_{int}}])}{dt} = \mu_{\mathrm{Ara}}([\mathrm{Arabinose_{ex}}] - [\mathrm{Arabinose_{int}}])$

ii. $\frac{d([\mathrm{Lactose_{int}}])}{dt} = \mu_{\mathrm{Lac}}([\mathrm{Lactose_{ex}}] - [\mathrm{Lactose_{int}}])$

iii. $\frac{d([\mathrm{Pbad_{mRNA}}])}{dt} = \alpha_{\mathrm{Leak\_Pbad}} + \left(\frac{k_{\mathrm{Pbad}} \times [\mathrm{Ara_{int}}]}{1+[\mathrm{Ara_{int}}]}\right) - \mathrm{HL_{mRNA\_Pbad}} \times [\mathrm{Pbad_{mRNA}}]$

iv. $\frac{d([\mathrm{Plac_{mRNA}}])}{dt} = \left(\frac{\alpha_{\mathrm{Plac}}}{1+[\mathrm{LacI}]^{\mathrm{Hill_{lac}}}}\right) + \alpha_{\mathrm{Leak\_Plac}} + \left(\frac{k_{\mathrm{Plac}} \times [\mathrm{Lac_{int}}]}{1+[\mathrm{Lac_{int}}]}\right) - \mathrm{HL_{mRNA\_Plac}} \times [\mathrm{Plac_{mRNA}}]$

v. $\frac{d([\mathrm{Ptet_{mRNA}}])}{dt} = \left(\frac{\alpha_{\mathrm{Ptet}}}{1+[\mathrm{tetR}]^{\mathrm{Hill_{tet}}}}\right) + \alpha_{\mathrm{Leak\_Ptet}} - \mathrm{HL_{mRNA\_Ptet}} \times [\mathrm{Ptet_{mRNA}}]$

vi. $\frac{d([\mathrm{mCherry}])}{dt} = \left(\mathrm{RBSmCherry} \times [\mathrm{Ptet_{mRNA}}] - \mathrm{HL_{ratio\_mCherry}} \times [\mathrm{mCherry}]\right)$

vii. $\frac{d([\mathrm{GFP}])}{dt} = \left(\mathrm{RBSGFP} \times [\mathrm{Plac_{mRNA}}] - \mathrm{HL_{ratio\_GFP}} \times [\mathrm{GFP}]\right)$

viii. $\frac{d([\mathrm{tetR}])}{dt} = \left(\mathrm{RBStetR} \times [\mathrm{Plac_{mRNA}}] - \mathrm{HL_{ratio\_tetR}} \times [\mathrm{tetR}]\right)$

ix. $\mathrm{LacI} = \mathrm{LacI_1} + \mathrm{LacI_2}$

x. $\frac{d([\mathrm{LacI_1}])}{dt} = \left(\mathrm{RBSLacI_1} \times [\mathrm{Pbad_{mRNA}}] - \mathrm{HL_{ratio\_LacI_1}} \times [\mathrm{LacI_1}]\right)$

xi. $\frac{d([\mathrm{LacI_2}])}{dt} = \left(\mathrm{RBSLacI_2} \times [\mathrm{Ptet_{mRNA}}] - \mathrm{HL_{ratio\_LacI_2}} \times [\mathrm{LacI_2}]\right)$

Our simulation had the following parameter values unless otherwise noted: HL_{mRNA_Pbad} ,HL_{mRNA_Plac}, HL_{mRNA_Ptet} = 1; α_{Pbad} = 0; α_{Ptet}, α_{Plac} = 0; α_{Pbad} = 200; $HL_{ratio\_mCherry}$, HL_{ratio_GFP}, HL_{ratio_TetR} HL_{ratio_LacI} = 3; RBS_{GFP}, $RBS_{mCherry}$, RBS_{etR}, RBS_{LacI}= 1; α_{Leak_Ptet}, α_{Leak_Plac}, α_{Leak_Pbad} = 1; k_{pbad}, k_{plac} = 50; k_{ptet} = 0; $Hill_{lac}$, $Hill_{tet}$ = 2. These parameters are based off of a calculation regime used in previous literature[8]. Additionally, Lactose_DoseConc, Arabinose_DoseConc = [50].

Figure 4 was generated by changing the RBS_{LacI} equal to 2.4. This value was chosen as a visually indicative change in the behavioral regime.

Figure 5 is the result of a two dimensional parameter sweep changing the RBS_{TetR} and RBS_{LacI} values from 1-10 by increments of 1. The simulations were run in series and the quantitative metrics describing the behavior for each RBS combination was assembled in an array. The array is visually represented by the heat contours shown in Figure 5.

Network Stochasticity for the Toggle Switch

We first simulated inducer stochasticity by incorporating a Gaussian kernel as a multiplier of the exponentially decaying internal inducer concentration. The results from this simulation are presented in Supplementary Figure S6. Due to the toggle switch's bistable nature, relatively small amounts of inducer stochasticity had negligible effects on the robotic emergent behavior[42].

To account for gene expression stochasticity, we modified our governing biochemical equations to include noise terms. These additions were based upon examples from literature[17] that augment the ODE's to include the stochastic terms ηR and ηP for transcriptional and translational noise, respectively.

In our continuous example, the terms ηR and ηP are defined as being random variables with a normal (Gaussian) distribution about a mean of zero. Within the model, the variance for ηR and ηP is defined as percentage of the mRNA or protein concentration at a given time step. For instance, for a given percentage, ρ, ηR and ηP are defined by S14 and S15 below, where $\mathbf{N}(\boldsymbol{\mu}, \boldsymbol{\nu})$ is a normal distribution as a function of the mean ($\boldsymbol{\mu}$) and the variance ($\boldsymbol{\nu}$).

$$\eta R = \mathbf{N}(0, (\rho * [\text{mRNA}])) \tag{S14}$$

$$\eta P = \mathbf{N}(0, (\rho * [\text{Protein}])) \tag{S15}$$

The random variables are then incorporated into the equations for translation S2 and transcription S3 to arrive at the stochastic versions of transcription and translation shown in equations S16 and S17, respectively.

$$\frac{d([\text{mRNA}])}{dt} = \left(\frac{\alpha}{1+[\text{RP}]^{H}}\right) + \alpha_{\text{Leak}} + \left(\frac{k \times [I_{int}]}{1+[I_{int}]}\right) - HL_{mRNA} \times [\text{mRNA}] + \eta R \tag{S16}$$

$$\frac{d([P])}{dt} = (\text{RBS} \times [\text{mRNA}] - HL_{ratio} \times [P]) + \eta P \tag{S17}$$

We simulated ρ = {0%, 1%, and 5%} for the transcription and translation of all mRNA and protein products. The results are shown in Figure S3 and Figures S7-S12.

Finally, in to consider environmental stochasticity we simulated the lactose and arabinose depots to appear at random locations. The results for four trial runs are shown in Figure S4.

Additional $P_{lux\text{-}\lambda}$ Operon

The simulations including the $P_{lux\text{-}\lambda}$ operon (Figures 6 and 7 in the main text) used a slightly different set of equations to accommodate the additional behavior.

i. $\frac{d([\text{Arabinose}_{int}])}{dt} = \mu_{Ara}([\text{Arabinose}_{ex}] - [\text{Arabinose}_{int}])$

ii. $\frac{d([Lactose_{int}])}{dt} = \mu_{Lac}([Lactose_{ex}] - [Lactose_{int}])$

iii. $\frac{d([Pbad_{mRNA}])}{dt} = \alpha_{Leak\_Pbad} + \left(\frac{k_{Pbad} \times [Ara_{int}]}{1+[Ara_{int}]}\right) - HL_{mRNA\_Pbad} \times [Pbad_{mRNA}]$

iv. $\frac{d([Plac_{mRNA}])}{dt} = \left(\frac{\alpha_{Plac}}{1+[LacI]^{Hill_{lac}}}\right) + \alpha_{Leak\_Plac} + \left(\frac{k_{Plac} \times [Lac_{int}]}{1+[Lac_{int}]}\right) - HL_{mRNA\_Plac} \times [Plac_{mRNA}]$

v. $\frac{d([Ptet_{mRNA}])}{dt} = \left(\frac{\alpha_{Ptet}}{1+[TetR]^{Hill_{tet}}}\right) + \alpha_{Leak\_Ptet} - HL_{mRNA\_Ptet} \times [Ptet_{mRNA}]$

vi. $\frac{d([mCherry_1])}{dt} = \left(RBSmCherry_1 \times [Ptet_{mRNA}] - HL_{ratio\_mCherry1} \times [mCherry_1]\right)$

vii. $\frac{d([GFP_1])}{dt} = \left(RBSGFP_1 \times [Plac_{mRNA}] - HL_{ratio\_GFP1} \times [GFP_1]\right)$

viii. $\frac{d([tetR])}{dt} = \left(RBSTetR \times [Plac_{mRNA}] - HL_{ratio\_tetR} \times [TetR]\right)$

ix. $LacI = LacI_1 + LacI_2$

x. $\frac{d([LacI_1])}{dt} = \left(RBSLacI_1 \times [Pbad_{mRNA}] - HL_{ratio\_LacI_1} \times [LacI_1]\right)$

xi. $\frac{d([LacI_2])}{dt} = \left(RBSLacI_2 \times [Ptet_{mRNA}] - HL_{ratio\_LacI_2} \times [LacI_2]\right)$

xii. $\frac{d([PLux-\lambda_{mRNA}])}{dt} = \left(\frac{\alpha_{PLux-\lambda}}{1+[cI]^{Hill_{cI}}}\right) + \alpha_{Leak\_PLux-\lambda} + \left(\frac{k_{PLux-\lambda} \times [AHL_{int}]}{1+[AHL_{int}]}\right) - HL_{mRNA\_PLux-\lambda} \times$
$[PLux - \lambda_{mRNA}]$

xiii. $\frac{d([AHL_{int}])}{dt} = \mu_{AHL}([AHL_{ex}] - [AHL_{int}])$

xiv. $\frac{d([mCherry_2])}{dt} = \left(RBSmCherry_2 \times [PLux - \lambda_{mRNA}] - HL_{ratio\_mCherry2} \times [mCherry_2]\right)$

xv. $\frac{d([GFP_2])}{dt} = \left(RBSGFP_2 \times [PLux - \lambda_{mRNA}] - HL_{ratio\_GFP2} \times [GFP_2]\right)$

xvi. $\frac{d([cI])}{dt} = \left(RBScI \times [PLux - \lambda_{mRNA}] - HL_{ratio\_cI} \times [cI]\right)$

xvii. $GFP = GFP_1 + GFP_2$

xviii. $mCherry = mCherry_1 + mCherry_2$

Furthermore, the additional circuit added the following modeling parameters: AHL_near = 2.0; AHL_far = 2.25; AHL_DoseConc = [130]; $HL_{mRNA\_PLux-\lambda} = 1$ $\alpha_{Plux-\lambda} = 0$; $\alpha_{Leak\_Plux-\lambda} = 1$; $k_{PLux-\lambda} = 100$; $HL_{ratio\_cI} = 1$; RBS_{GFP_2}, $RBS_{mCherry\_2} = 4$.

For Figure 7 of the main text, we varied the RBScI value from 0 to 1 and observed where regime shifts occurred. We visually inspected the simulation results and found areas of regime bifurcations. The selected regimes are indicative of the major visually observed behavioral shifts.

REFERENCES

1. Tlaskalova-Hogenova, H. *et al.* Commensal bacteria (normal microflora), mucosal immunity and chronic inflammatory and autoimmune diseases. *Immunol. Lett.* **93**, 97-108, doi:10.1016/j.imlet.2004.02.005 (2004).

2. Walter, J. & Ley, R. The human gut microbiome: ecology and recent evolutionary changes. *Annu. Rev. Microbiol.* **65**, 411-429, doi:10.1146/annurev-micro-090110-102830 (2011).

3. Liu, L. *et al.* The human microbiome: a hot spot of microbial horizontal gene transfer. *Genomics* **100**, 265-270, doi:10.1016/j.ygeno.2012.07.012 (2012).

4. Brenner, K., You, L. & Arnold, F. H. Engineering microbial consortia: a new frontier in synthetic biology. *Trends Biotechnol.* **26**, 483-489, doi:10.1016/j.tibtech.2008.05.004 (2008).

5. Brenner, K. & Arnold, F. H. Self-organization, layered structure, and aggregation enhance persistence of a synthetic biofilm consortium. *PLoS One* **6**, e16791, doi:10.1371/journal.pone.0016791 (2011).

6. Gardner, T. S., Cantor, C. R. & Collins, J. J. Construction of a genetic toggle switch in Escherichia coli. *Nature* **403**, 339-342, doi:10.1038/35002131 (2000).

7. Elowitz, M. B. & Leibler, S. A synthetic oscillatory network of transcriptional regulators. *Nature* **403**, 335-338, doi:10.1038/35002125 (2000).

8. Garcia-Ojalvo, J., Elowitz, M. B. & Strogatz, S. H. Modeling a synthetic multicellular clock: repressilators coupled by quorum sensing. *Proc. Natl. Acad. Sci. U. S. A.* **101**, 10955-10960, doi:10.1073/pnas.0307095101 (2004).

9. Hasty, J., Isaacs, F., Dolnik, M., McMillen, D. & Collins, J. J. Designer gene networks: Towards fundamental cellular control. *Chaos* **11**, 207-220, doi:10.1063/1.1345702 (2001).

10. Elowitz, M. B., Levine, A. J., Siggia, E. D. & Swain, P. S. Stochastic gene expression in a single cell. *Science* **297**, 1183-1186, doi:10.1126/science.1070919 (2002).

11. Gillespie, D. T. A General Method for Numerically Simulating the Stochastic Time Evolution of Coupled Chemical Reactions. *JCoPh* **22**, 403-434 (1976).

12. MacDonald, J. T., Barnes, C., Kitney, R. I., Freemont, P. S. & Stan, G. B. Computational design approaches and tools for synthetic biology. *Integr. Biol. (Camb.)* **3**, 97-108, doi:10.1039/c0ib00077a (2011).

13. Wilkinson, D. J. Stochastic modelling for quantitative description of heterogeneous biological systems. *Nat. Rev. Genet.* **10**, 122-133, doi:10.1038/nrg2509 (2009).

14. Tian, T. & Burrage, K. Stochastic models for regulatory networks of the genetic toggle switch. *Proc. Natl. Acad. Sci. U. S. A.* **103**, 8372-8377, doi:10.1073/pnas.0507818103 (2006).

15. Rudge, T. J., Steiner, P. J., Phillips, A. & Haseloff, J. Computational modeling of synthetic microbial biofilms. *ACS Synth Biol* **1**, 345-352, doi:10.1021/sb300031n (2012).

16. Raj, A. & van Oudenaarden, A. Nature, Nurture, or Chance: Stochastic Gene Expression and Its Consequences. *Cell* **135**, 216-226, doi:http://dx.doi.org/10.1016/j.cell.2008.09.050 (2008).

17. Ozbudak, E. M., Thattai, M., Kurtser, I., Grossman, A. D. & van Oudenaarden, A. Regulation of noise in the expression of a single gene. *Nat. Genet.* **31**, 69-73 (2002).

18. Karlebach, G. & Shamir, R. Modelling and analysis of gene regulatory networks. *Nat. Rev. Mol. Cell Biol.* **9**, 770-780, doi:10.1038/nrm2503 (2008).

19. Buse, O., Perez, R. & Kuznetsov, A. Dynamical properties of the repressilator model. *Phys. Rev. E Stat. Nonlin. Soft Matter Phys.* **81**, 066206 (2010).

20. Vilar, J. M., Guet, C. C. & Leibler, S. Modeling network dynamics: the lac operon, a case study. *J. Cell Biol.* **161**, 471-476, doi:10.1083/jcb.200301125 (2003).

21. Brewster, R. C., Jones, D. L. & Phillips, R. Tuning Promoter Strength through RNA Polymerase Binding Site Design in *E. Coli. PLoS Comput Biol* **8**, e1002811, doi:10.1371/journal.pcbi.1002811 (2012).

22. Lopez, P. J., Marchand, I., Yarchuk, O. & Dreyfus, M. Translation inhibitors stabilize Escherichia coli mRNAs independently of ribosome protection. *Proc. Natl. Acad. Sci. U. S. A.* **95**, 6067-6072 (1998).

23. Isaacs, F. J. *et al.* Engineered riboregulators enable post-transcriptional control of gene expression. *Nat. Biotechnol.* **22**, 841-847, doi:10.1038/nbt986 (2004).

24. Cameron, D. E., Bashor, C. J. & Collins, J. J. A brief history of synthetic biology. *Nat. Rev. Microbiol.* **12**, 381-390, doi:10.1038/nrmicro3239 (2014).

25. Mee, M. T. & Wang, H. H. Engineering ecosystems and synthetic ecologies. *Mol Biosyst* **8**, 2470-2483, doi:10.1039/c2mb25133g (2012).

26. Esvelt, K. M. & Wang, H. H. Genome-scale engineering for systems and synthetic biology. *Mol. Syst. Biol.* **9**, 641, doi:10.1038/msb.2012.66 (2013).

27. Brophy, J. A. & Voigt, C. A. Principles of genetic circuit design. *Nat. Methods* **11**, 508-520, doi:10.1038/nmeth.2926 (2014).

28. Kobayashi, H. *et al.* Programmable cells: interfacing natural and engineered gene networks. *Proc. Natl. Acad. Sci. U. S. A.* **101**, 8414-8419, doi:10.1073/pnas.0402940101 (2004).

29. Huang, H. & Densmore, D. Integration of microfluidics into the synthetic biology design flow. *Lab Chip* **14**, 3459-3474, doi:10.1039/c4lc00509k (2014).

30. Lee, K. S., Boccazzi, P., Sinskey, A. J. & Ram, R. J. Microfluidic chemostat and turbidostat with flow rate, oxygen, and temperature control for dynamic continuous culture. *Lab Chip* **11**, 1730-1739, doi:10.1039/c1lc20019d (2011).

31. Groisman, A. *et al.* A microfluidic chemostat for experiments with bacterial and yeast cells. *Nat. Methods* **2**, 685-689, doi:10.1038/nmeth784 (2005).

32. Bennett, M. R. & Hasty, J. Microfluidic devices for measuring gene network dynamics in single cells. *Nat. Rev. Genet.* **10**, 628-638, doi:10.1038/nrg2625 (2009).

33. Ghosh, K. K. *et al.* Miniaturized integration of a fluorescence microscope. *Nat. Methods* **8**, 871-878, doi:10.1038/nmeth.1694 (2011).

34. Cianci, C., Raemy, X., Pugh, J. & Martinoli, A. in *Swarm Robotics* Vol. 4433 *Lecture Notes in Computer Science* (eds Erol Şahin, WilliamM Spears, & AlanF T. Winfield) Ch. 7, 103-115 (Springer Berlin Heidelberg, 2007).

35. Braitenberg, V. *Vehicles: Experiments in Synthetic Psychology*. First edn, (MIT University Press, 1984).

36. Danino, T., Mondragon-Palomino, O., Tsimring, L. & Hasty, J. A synchronized quorum of genetic clocks. *Nature* **463**, 326-330, doi:10.1038/nature08753 (2010).

37. Balagadde, F. K. *et al.* A synthetic Escherichia coli predator-prey ecosystem. *Mol. Syst. Biol.* **4**, 187, doi:10.1038/msb.2008.24 (2008).

38. Hong, S. H. *et al.* Synthetic quorum-sensing circuit to control consortial biofilm formation and dispersal in a microfluidic device. *Nat Commun* **3**, 613, doi:10.1038/ncomms1616 (2012).

39. Keasling, J. D. Gene-expression tools for the metabolic engineering of bacteria. *Trends Biotechnol.* **17**, 452-460, doi:http://dx.doi.org/10.1016/S0167-7799(99)01376-1 (1999).

40. Wong, P., Gladney, S. & Keasling, J. D. Mathematical model of the lac operon: inducer exclusion, catabolite repression, and diauxic growth on glucose and lactose. *Biotechnol. Prog.* **13**, 132-143, doi:10.1021/bp970003o (1997).

41. Shea, M. A. & Ackers, G. K. The OR control system of bacteriophage lambda. A physical-chemical model for gene regulation. *J. Mol. Biol.* **181**, 211-230, doi:http://dx.doi.org/10.1016/0022-2836(85)90086-5 (1985).

42. Tian, T. & Burrage, K. Stochastic models for regulatory networks of the genetic toggle switch. *Proceedings of the National Academy of Sciences* **103**, 8372-8377, doi:10.1073/pnas.0507818103 (2006).
